# Genetic characterization of root architectural traits in barley (*Hordeum vulgare* L.) using SNP markers

**DOI:** 10.3389/fpls.2023.1265925

**Published:** 2023-10-04

**Authors:** M. Q. U. Farooqi, David Moody, Guihua Bai, Amy Bernardo, Paul St. Amand, Art J. Diggle, Zed Rengel

**Affiliations:** ^1^ UWA School of Agriculture and Environment, The University of Western Australia, Perth, WA, Australia; ^2^ InterGrain, Bibra Lake, WA, Australia; ^3^ Hard Winter Wheat Genetics Research Unit, USDA-ARS, Manhattan, KS, United States; ^4^ Department of Primary Industries and Regional Development, South Perth, WA, Australia

**Keywords:** barley, genotyping-by-sequencing, SNP markers, association mapping, root traits

## Abstract

Increasing attention is paid to providing new tools to breeders for targeted breeding for specific root traits that are beneficial in low-fertility, drying soils; however, such information is not available for barley (*Hordeum vulgare* L.). A panel of 191 barley accessions (originating from Australia, Europe, and Africa) was phenotyped for 26 root and shoot traits using the semi-hydroponic system and genotyped using 21 062 high-quality single nucleotide polymorphism (SNP) markers generated by genotyping-by-sequencing (GBS). The population structure analysis of the barley panel identified six distinct groups. We detected 1199 significant (*P<*0.001) marker-trait associations (MTAs) with r^2^ values up to 0.41. The strongest MTAs were found for root diameter in the top 20 cm and the longest root length. Based on the physical locations of these MTAs in the barley reference genome, we identified 37 putative QTLs for the root traits, and three QTLs for shoot traits, with nine QTLs located in the same physical regions. The genomic region 640-653 Mb on chromosome 7H was significant for five root length-related traits, where 440 annotated genes were located. The putative QTLs for various root traits identified in this study may be useful for genetic improvement regarding the adaptation of new barley cultivars to suboptimal environments and abiotic stresses.

## 1 Introduction

Barley (*Hordeum vulgare* L.) is the fourth most widely grown cereal crop worldwide after maize, rice and wheat (Schulte et al., 2009). Barley is increasingly used as an important source of nutrition for humans, although it has been used traditionally as animal feed and in the brewing industry. Intensive breeding for more than a century has resulted in a wide range of barley genotypes that are well adapted to various climatic conditions, with barley being considered a model species for temperate cereals (Badr et al., 2000; Schulte et al., 2009). However, genetic diversity is presumed to have declined in modern barley cultivars, forcing the introduction of exotic germplasm as one of the major genetic resources in innovative modern breeding programs (Ellis et al., 2000).

Roots are essential to plants for a variety of processes, including uptake and storage of water and nutrients, and support and anchoring of above-ground plant parts (Aroca et al., 2005). Root system architecture (RSA) plays an important role in soil exploration and nutrient accumulation (Rose et al., 2009; Chen et al., 2016). The adaptive characteristics of roots (especially in cereals) to withstand harsh environments have been investigated only to a limited extent due to (i) lack of knowledge on root system growth and functioning, (ii) difficulties in measuring relevant traits, and (iii) non-availability of an efficient root screening method (Richards et al., 2002). Compared to other cereals, barley has a higher degree of adaptability under different types of stresses (e.g. heat and drought), likely due to early root development and an extensive root system (Maurer et al., 2018). In addition, barley roots can grow to 2-meter depths in soils such as loams or deep sands (Gahoonia et al., 2001).

Molecular markers have been used widely to dissect complex traits in different species (Mishra et al., 2014; Xue et al., 2019; Mehrabi et al., 2020; Oyiga et al., 2020; Salarpour et al., 2020). Advanced genotyping technologies such as genotyping-by-sequencing (GBS) are powerful methods to discover molecular markers using next-generation-sequencing (NGS) and allow rapid identification of genomic regions associated with the traits of interest (e.g. Elshire et al., 2011; Mehrabi et al., 2020; Oyiga et al., 2020). Recently, high-throughput NGS-based single nucleotide polymorphisms (SNPs) have been found to be efficient markers for genetic studies and genomic selection in breeding programs because of the low cost of acquiring a large number of markers and their co-dominant nature (Close et al., 2009). The first set of barley SNP markers was developed in elite European varieties (Kota et al., 2001). Chloupek et al. (2006) used SNP markers to study root system size in a panel of diverse genotypes and a double haploid (DH) population of barley. Naz et al. (2014) and Reinert et al. (2016) found important quantitative trait loci (QTLs) related to root and shoot traits in the different sets of barley germplasm.

Association genetics offers a powerful approach to identifying genetic variants that control complex traits and can be used to explore genetic variations associated with various traits across genotypes (Zhou et al., 2016; Mehrabi et al., 2020; Salarpour et al., 2020). Recently, Reinert et al. (2016) reported a GWAS using 5892 SNP markers and identifying QTLs that control barley root traits. Using the same strategy, Oyiga et al. (2020) found 11 QTLs associated with barley root architectural and anatomical responses to drought stress. However, the power and resolution of GWAS depends strongly on the extent of linkage disequilibrium (LD) across the whole genome of tested cultivars (Gupta et al., 2005). Various factors can affect LD, including allele frequency, population structure, mating system and admixture (Flint-Garcia et al., 2003). Therefore, a thorough understanding of population structure and LD patterns across the genome is critical to assembling a diversity panel for association studies.

We used an association panel containing 191 barley genotypes, mainly advanced breeding lines and key parental germplasm lines developed in the InterGrain Pty Ltd (Bibra Lake, Western Australia) barley breeding program between 2011 and 2015. The panel was phenotyped for root traits and genotyped using GBS-SNP markers. The objectives of this study were to (i) evaluate genetic variation in root traits in the panel, (ii) estimate the LD decay, and (iii) identify the major genetic regions responsible for various barley root traits that could be used in breeding for improved barley root architecture.

## 2 Materials and methods

### 2.1 Plant materials and phenotyping

The 191 genotypes with 101 different pedigrees and of different origins (Australia, Europe and Ethiopia), with most (126 genotypes) being InterGrain advanced breeding lines, were used in this study (**Supplementary Table 1**). The phenotyping experiment was performed in a glasshouse at The University of Western Australia using an established 1.0-m-tall semi-hydroponic system (Chen et al., 2011a; Chen et al., 2011b; Chen et al., 2012) and a randomized complete block design with three biological replicates and two plants per replicate. The phenotypic data are reported elsewhere (Wang et al., 2021). Shoot height and leaflet number in each plant were determined at harvest. After harvesting, shoots and roots were separated, and root tissues were subsampled further by cutting into 20-cm sections (starting from the crown) to determine root morphology as described previously (Chen et al., 2011a; Chen et al., 2011b; Chen et al., 2012).

Root surface area, total root length, root volume, and average root diameter were measured based on root images of each root section using WinRHIZO software (Regent Instruments, Quebec, Canada). Root lengths were determined for different root diameter classes (in mm):<0.06, 0.06–0.065, 0.065–0.70, 0.70–0.075, 0.075–0.085, 0.085–0.10, 0.10–0.15, 0.15–0.25, 0.25–0.40, 0.40–0.65 and >0.65. Given the variable root lengths in various diameter classes (with almost no root length in some classes), all root lengths were pooled into three representative diameter classes: thin (<0.075 mm), medium (0.075-0.25 mm) and thick (>0.25 mm).

In addition to the parameters measured, root length ratio (RLR) and specific root length (SRL) were calculated (**Table 1**) for marker-trait association (MTA) analysis as follows:

**Table 1:** Root phenotypic parameters and their codes.

Parameter	Code
Total Root Length	TRL
Longest Root Length	LRL
Root Length in 20-100 cm	RL_lower
Root Length in the top part (0-20 cm depth)	RL_top
Root Length below top part (20-40 cm depth)	RL_20
Root Length below 40 cm depth	RL_40
Root Length Ratio (root length 0-20 cm depth/root length below 20 cm depth)	RLR
Specific Root Length (length/dry matter mass)	SRL
average Root Diameter	RD
root Diameter Class Length thin (roots with<0.075 mm diameter)	DCL_thin
root Diameter Class Length medium (roots with 0.075-0.25 mm diameter)	DCL_med
root Diameter Class Length thick (roots with >0.25 mm diameter)	DCL_thick
Root Diameter (0-20 cm depth)	RD_top
Root Diameter (20-40 cm depth)	RD_20
Root Diameter (below 40 cm depth)	RD_40
total Root Area	RA
total Root Volume	RV
total Root dry Biomass	RB
Root-to-Shoot dry mass ratio	R/S
Root dry Biomass (0-20 cm depth)	RB_top
Root dry Biomass (20-40 cm depth)	RB_20
Root dry Biomass (below 40 cm depth)	RB_40
Lateral Root Number	LRN
Shoot Biomass	SB
Shoot Height	SH
Tiller number	Till


RLR=root length at 0-20 cm depth/root length below 20 cm depth

$$\text{SRL}=\text{root length}/\text{root drymass }({\text{m g}}^{-1})$$

### 2.2 DNA extraction and SNP assays

Genomic DNA of the barley samples was extracted from fresh leaf tissue of 2-week-old seedlings as described by Kotchoni and Gachomo (2009). A GBS library was constructed for 191 barley DNA samples at Kansas State University, Manhattan, KS, USA, following Poland et al. (2012). In brief, DNA samples were digested with the *PstI*-HF (high fidelity) and *MspI* restriction enzymes (New England BioLabs Inc., Ipswich, MA, USA), and ligated to barcoded adapters and a common ‘Y’ adapter using T4 DNA ligase (New England BioLabs Inc.). All ligation products in the two 96-well plates were pooled and cleaned up using a QIAquick PCR Purification Kit (Qiagen Inc., Valencia, CA, USA). Primers complementary to both adapters were used for PCR. The PCR products were then cleaned again using the QIAquick PCR Purification Kit and size-selected for a range of 250-300 base-pairs (bp) in an E-gel system (Life Technologies Inc., Carlsbad, CA, USA). DNA concentration was estimated using a Qubit 2.0 fluorometer and a dsDNA HS Assay kit (Life Technologies Inc.). The size-selected library was sequenced on an Ion Proton semiconductor sequencer (Life Technologies Inc.).

SNPs were called using both the reference-based pipeline (Li et al., 2009) and the Universal Network-Enabled Analysis Kit (UNEAK) pipeline in Trait Analysis by aSSociation, Evolution and Linkage (TASSEL) v.5 for SNP/variant discovery (Bradbury et al., 2007). Raw sequence reads from the Ion Torrent system with variable lengths were supplemented with poly-A at their 3’ ends to ensure all reads had lengths of at least 64 bp as required by the TASSEL software. TASSEL sorted all the reads by barcodes and auto-trimmed them to 64 bases. Bi-allelic SNPs were determined by querying the filtered tags for pairs of sequences (Poland et al., 2012) if they differed in only one or two SNPs. SNPs called by two pipelines were merged, and duplicated SNPs were removed. Only the SNPs that were present in at least 80% of the genotypes in the population were used for further analysis. SNPs with a minor allele frequency<0.05 and heterozygous in >10% of the accessions were excluded from the analysis (**Supplementary Figure 1**).

### 2.3 Population structure and linkage disequilibrium

Population structure of the bi-parental multi-locus genotypes was analyzed using the Bayesian clustering procedure implemented in STRUCTURE 2.3.4 (Falush et al., 2003). Initial STRUCTURE runs were carried out with a length of 100 000 burn-in-periods and 10 000 MCMC (Markov Chain Monte Carlo) iterations by increasing K-values from 1 to 10. The most probable number of groups was determined by plotting the estimated likelihood values LnP(D) obtained from STRUCTURE runs against five repetitions of K values. LnP(D) is the log likelihood of observed genotype distribution in K clusters as calculated by STRUCTURE. The K value best describing the population structure was based on the criteria of maximizing ΔK (Evanno et al., 2005) using Structure Harvester v.0.6.94 (Earl and VonHoldt, 2012).

A proportion of the phenotypic variation explained by the model was assessed using correlation coefficients (r). Principal component analysis (PCA) was done by using “FactoMineR” in R v.4.1.2 to evaluate the population structure, whereas cluster analysis was conducted using the kinship matrix elements as similarities by utilizing all given root and shoot traits data. The resulting output was visualized through the use of kinship matrix, with the aim of uncovering population information using Genome Association and Prediction Integrated Tool (GAPIT) (Lipka et al., 2012). To assess the accuracy in genotypic selection, an imputation was done using LD-kNNi by introducing *k*-nearest neighbor to evaluate the SNPs having strongest LD in genomic data (Money et al., 2015). In LD-kNNi, it is not necessary to refer to physical linkage, but rather to the correlation between any two SNPs in the dataset. The LD pattern across seven chromosomes of barley was investigated using TASSEL 5.2.44. The outputs from TASSEL (using sliding window of 50 SNPs and maf = 0.05) were used to generate LD decay plots using R. The LD decay explained by r^2^ was determined using 21 062 high-quality GBS-SNPs. The extent and distribution of LD were visualized by plotting intra-chromosomal r^2^ values (at *p<*0.001) against the physical distance in base pairs using 1000 permutations. The estimation of LD decay using LOESS (locally estimated scatterplot smoothing) involved the determination of genetic distance at which the LOESS curve initially intersects the baseline r^2^ value. The average LD decay of the panel was utilized to determine the point of intersection between the LOESS curve and the critical r^2^. Unlinked r^2^ estimates were square-root transformed to approximate a normally distributed random variable, and the parametric 95^th^ percentile of that distribution was taken as a critical value of r^2^ indicated in blue lines (**Supplementary Figure 4**), beyond which LD was likely caused by genetic linkage (Breseghello and Sorrells, 2006).

### 2.4 Marker-trait association

Marker-trait association analysis was done by utilizing various GWAS (GLM, MLM, MLMM, SUPER, and BLINK) with 26 root and shoot traits (**Table 1**) using R package GAPIT v.3.0 (Lipka et al., 2012). In comparison, Bayesian information criterion (BIC) and Linkage-disequilibrium Iteratively Nested Keyway (BLINK) model was better fit as it has demonstrated a reduced incidence of false positives, improved detection of true positives, and the ability to handle extensive datasets. BLINK is an improved model version of Fixed and Random Model Circulating Probability Unification (FarmCPU) and is statistically powerful and efficient in identifying significant SNPs associated with a trait of importance; hence, BLINK has been used in the present study. Using the panel of 21 062 SNP markers, we estimated random and fixed effects in order to reduce the rate of false positives in the BLINK model (Huang et al., 2019). The optimization was performed using BIC, which is twice the negative log likelihood plus the three penalties on number of parameters, using the equation BIC = -2 ln(L) + 2 k ln(n), where lnL is log likelihood, k is the number of pseudo QTNs (quantitative trait nucleotides), and n is the number of individuals. To reduce false MTAs due to population structure and relatedness, the mixed model incorporating principal components and a kinship matrix was used in the R package GAPIT v.3.0 (Lipka et al., 2012). Model correction for population structure and cryptic relatedness between lines was based on a compressed Efficient Mixed-Model Association (EMMA) kinship matrix with Q-values from STRUCTURE K=6 included as an efficient fixed effect. Manhattan plots showing positions of associated markers across the genome were constructed for each trait using the R package CMplot (Yin, 2018). The false discovery rate (FDR)-adjusted p-values used in GAPIT were found to be highly stringent. The FDR correction was performed using the Benjamini–Hochberg method to obtain a q-value (FDR-adjusted p-value) (Benjamini and Hochberg, 1995). A threshold of *q ≤*0.05 was used to claim significant MTAs, after Bonferroni multiple test correction which corresponds to *p ≤*0.001 significant level used in BLINK model. Markers were clustered into one locus based on LD decay (2.97 Mb), and the locus was categorized as a QTL.

GBS-SNPs closely associated with QTLs were mapped to physical positions (Mb) by blasting the marker sequences through the *GrainGenes* database against the barley reference genome assembly *Hordeum vulgare* (Barley Morex V3) developed by the International Barley Genome Sequencing Consortium (Mascher et al., 2021). Furthermore, MapChart v.2.0 (Voorrips, 2002) was used to depict the linkage map and detected QTLs based on their physical positions (Mb). The functional annotation of potential candidate genes given in **Table 2** was retrieved from the online tool BARLEYMAP (http://floresta.eead.csic.es/barleymap/) (Cantalapiedra et al., 2015).

**Table 2:** Selected single nucleotide polymorphism (SNP) markers showing the strongest significant marker-trait associations with various root and shoot traits in the barley association panel.

Marker ID	Alleles	Chromosome	Physical position (Mb)	Trait *	–log10 (p-value)	GBS candidate genes	Functional annotation
S3H_634086093	C/T	3H	634.09	RD_top	17.88	HORVU3Hr1G090970	IPR004159 (S-adenosyl-L-methionine-dependent methyltransferases superfamily protein)
S3H_634086093	C/T	3H	634.09	RD_20	14.24	HORVU3Hr1G090970	IPR004159 (S-adenosyl-L-methionine-dependent methyltransferases superfamily protein)
S4H_597515417	A/G	4H	597.52	RD_40	11.76	HORVU4Hr1G075250	IPR011598 (Transcription factor ILI6)
S5H_218040048	G/C	5H	218.04	RD_top	11.66	HORVU5Hr1G033010	IPR000095 (ROP-interactive CRIB motif-containing protein)
S5H_667512618	T/G	5H	667.51	RB_20	10.14	HORVU5Hr1G124820	IPR002168 (alpha/beta-hydrolases superfamily protein)
UNEAK-GBS-SNP-1074	C/A	2H	243.69	RD_20	9.34	HORVU2Hr1G036320	IPR000719 (WRKY family transcription factor family protein)
UNEAK-GBS-SNP-4246	T/A	6H	23.53	RB	8.67	HORVU6Hr1G012240	IPR000014 (protein kinase family protein)
UNEAK-GBS-SNP-859	G/A	2H	45.70	R/S	8.47	HORVU2Hr1G017940	IPR029688 (Interactor of constitutive active ROPs 3)
S3H_2360174	A/G	3H	2.36	RD	8.42	HORVU3Hr1G001020	IPR010369 (Domain of unknown function)
UNEAK-GBS-SNP-3537	G/A	5H	301.68	RL_top	8.36	HORVU5Hr1G040540	IPR002885 (Pentatricopeptide repeat-containing protein)
UNEAK-GBS-SNP-859	G/A	2H	45.70	LRN	8.35	HORVU2Hr1G017940	IPR029688 (Interactor of constitutive active ROPs 3)
S5H_645158922	T/C	5H	645.16	RD_40	7.75	HORVU5Hr1G114950	IPR000719 (receptor kinase 3)
S2H_617361612	A/G	2H	617.36	RB_top	7.51	HORVU2Hr1G075240	IPR000595 (Thioredoxin superfamily protein)
UNEAK-GBS-SNP-4246	T/A	6H	23.53	RB_40	7.48	HORVU6Hr1G012240	IPR000014 (protein kinase family protein)
UNEAK-GBS-SNP-2556	G/A	4H	65.62	RLR	7.35	HORVU4Hr1G016210	IPR000719 (receptor kinase 3)
S1H_162694706	T/C	1H	162.69	RB_20	7.20	HORVU1Hr1G080950	IPR002182 (Glycogen synthase)
UNEAK-GBS-SNP-1552	T/C	2H	730.57	RD	6.93	HORVU2Hr1G113930	IPR000209 (Subtilisin-like protease)
S7H_648775405	G/T	7H	648.78	SH	6.66	HORVU7Hr1G033820	IPR002213 (Auxin efflux carrier family protein)
S2H_727648550	C/T	2H	727.65	RB_40	5.99	HORVU2Hr1G113210	IPR002182 (Disease resistance protein)
S5H_311285005	G/T	5H	311.29	SH	5.87	HORVU5Hr1G041250	IPR007315 (GPI mannosyltransferase 2)
UNEAK-GBS-SNP-1206	G/C	2H	453.16	RLR	5.20	HORVU2Hr1G065760	IPR002921 (lipase class 3 family protein)
S5H_526798135	C/A	5H	526.80	RL_top	5.14	HORVU5Hr1G070010	IPR007052 (HSP20-like chaperone superfamily protein isoform 1)
S1H_498665649	G/A	1H	498.67	DCL_thin	4.34	HORVU1Hr1G072690	IPR000477 (Telomerase reverse transcriptase)
S5H_69488625	T/G	5H	69.49	RB	4.31	HORVU5Hr1G017770	IPR002035 (transport protein SEC24)
UNEAK-GBS-SNP-1803	C/T	3H	104.25	R/S	4.27	HORVU3Hr1G026070	IPR000014 (protein kinase family protein)
S2H_602228561	G/A	2H	602.23	DCL_thick	4.21	HORVU2Hr1G082860	IPR029688 (Interactor of constitutive active ROPs 3)
UNEAK-GBS-SNP-2925	A/G	4H	504.39	DCL_thick	4.21	HORVU4Hr1G060180	IPR002129 (L-tyrosine decarboxylase)
S1H_498516564	C/A	1H	498.52	DCL_thin	4.19	HORVU1Hr1G072620	1PR0016111 (Leucine-rich repeat (LRR) family protein)
S1H_498665554	C/T	1H	498.67	DCL_thin	4.10	HORVU1Hr1G072690	IPR000477 (Telomerase reverse transcriptase)
UNEAK-GBS-SNP-2469	A/C	4H	12.60	Till	4.04	HORVU4Hr1G005610	IPR000095 (ROP-interactive CRIB motif-containing protein)
S7H_157328599	G/T	7H	157.33	Till	4.04	HORVU7Hr1G047230	IPR005333 (TCP family transcription factor 4)
S1H_498665649	G/A	1H	498.67	LRL	4.04	HORVU1Hr1G072690	IPR000477 (Telomerase reverse transcriptase)
S1H_498516564	C/A	1H	498.52	LRL	3.93	HORVU1Hr1G072620	1PR0016111 (Leucine-rich repeat (LRR) family protein)
UNEAK-GBS-SNP-1526	T/G	2H	723.51	RL_40	3.85	HORVU2Hr1G111620	IPR001611 (Leucine-rich repeat (LRR) family protein)
UNEAK-GBS-SNP-1761	C/T	3H	63.36	RL_40	3.81	HORVU3Hr1G020390	IPR010369 (Domain of unknown function)
S2H_45699171	G/A	2H	45.70	RB_top	3.76	HORVU2Hr1G017950	IPR000477 (Telomerase reverse transcriptase)
S4H_36522655	C/T	4H	36.52	RA	3.74	HORVU4Hr1G011620	IPR002213 (UDP-glycosyltransferase superfamily protein)
UNEAK-GBS-SNP-4951	G/A	6H	513.97	RL_lower	3.70	HORVU6Hr1G074430	IPR008030 (high chlorophyll fluorescence phenotype 173)
UNEAK-GBS-SNP-4951	G/A	6H	513.97	SB	3.68	HORVU6Hr1G074430	IPR008030 (high chlorophyll fluorescence phenotype 173)
UNEAK-GBS-SNP-98	C/T	1H	27.41	RA	3.55	HORVU1Hr1G011360	IPR029688 (Interactor of constitutive active ROPs 3)
S3H_686750162	C/T	3H	686.75	SRL	3.44	HORVU3Hr1G112690	IPR001611 (Leucine-rich repeat (LRR) family protein)
S6H_532368578	C/T	6H	532.37	SRL	3.44	HORVU6Hr1G077840	IPR002182 (Disease resistance protein)
S4H_36522655	C/T	4H	36.52	RV	3.40	HORVU4Hr1G011620	IPR002213 (UDP-glycosyltransferase superfamily protein)
UNEAK-GBS-SNP-4345	T/C	6H	59.77	SB	3.38	HORVU6Hr1G020750	IPR002921 (lipase class 3 family protein)
S1H_475311368	T/G	1H	475.31	LRN	3.36	HORVU1Hr1G067000	IPR001810 (F-box domain containing protein)
S5H_608844957	A/G	5H	608.84	RV	3.28	HORVU5Hr1G098780	IPR000719 (receptor kinase 3)
S7H_591555976	A/G	7H	591.56	RL_20	3.25	HORVU7Hr1G097250	IPR000014 (protein kinase family protein)
S7H_591555977	C/A	7H	591.56	RL_20	3.25	HORVU7Hr1G097250	IPR000014 (protein kinase family protein)
UNEAK-GBS-SNP-1761	C/T	3H	63.36	TRL	3.24	HORVU3Hr1G020390	IPR010369 (Domain of unknown function)
S5H_667065218	C/T	5H	667.07	TRL	3.23	HORVU5Hr1G124680	IPR000719 (receptor kinase 3)
UNEAK-GBS-SNP-914	G/A	2H	76.36	DCL_med	3.16	HORVU2Hr1G024880	IPR000778 (respiratory burst oxidase homolog B)
S3H_686750146	G/A	3H	686.75	DCL_med	3.16	HORVU3Hr1G112690	IPR001611 (Leucine-rich repeat (LRR) family protein)
S4H_416803319	G/T	4H	416.80	RL_lower	3.04	HORVU4Hr1G050970	IPR000243 (Proteasome subunit beta type-5-A)

*The trait codes are listed in **Table 1**.

## 3 Results

### 3.1 Evaluation of root traits

The statistical evaluation of the phenotypic data from 189 accessions (excluding the lines IG15RT_0166 and IG15RT_0171 that failed to germinate during phenotyping) was presented elsewhere (Wang et al., 2021). Out of 26 root and shoot traits evaluated, 16 traits (13 root and three shoot traits) showed highly significant differences among genotypes, with coefficient of variation (CV) values ≥0.25 (Wang et al., 2021). For example, the average length of longest root was 113 ± 15 cm (mean ± SD), ranging from 60 to 158 cm across genotypes.

### 3.2 Population structure, principal components, and genetic relatedness

The panel of 191 barley germplasm lines generated more than 169 million sequence reads and 3 407 301 total SNP datapoints. Apart from 12 samples that generated high missing data rates due to poor DNA quality, all other samples produced high quality GBS-SNPs (5951 from UNEAK and 15 111 from the reference-based pipeline) with ≤20% missing data and were used for further analysis.

**Figure 1:**
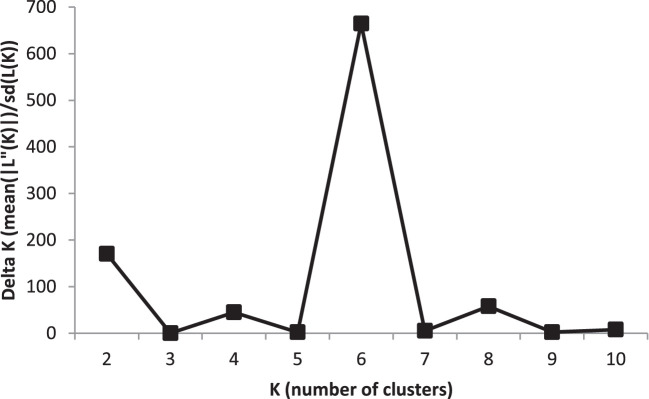
An estimate of the most probable number of clusters based on ΔK in five iteration runs using GBS-SNP markers in the barley association panel.

To analyse population structure, the clustering parameter K (1 to 10) was used to group genotypes based on genetic relationships from GBS-SNPs. The most probable number of structured subpopulations (K value) and the optimal K value were found by graphing ΔK against K, which showed a sharp peak at K = 6. Hence, six distinct groups were identified with a mean log likelihood [LnP(D)] value of -165 938, ranging from -212 480 (K = 1) to -168 367 (K = 6), with the highest ΔK value at K = 6 (**Figure 1**; **Supplementary Figure 2**; **Supplementary Table 1**). The LnP(D) values increased gradually from K=1 with some variation among K values, with K = 6 being the predicted number of major clusters in the barley panel. With the rise in K to the optimal K value, a steady improvement in the evaluated Ln P(D) was seen, indicating that six subpopulations contained all 191 genotypes with the greatest probability. These six sub-populations had the following numbers of genotypes: G1 (21), G2 (24), G3 (13), G4 (74), G5 (19), and G6 (40) (**Supplementary Figure 2**; **Supplementary Table 1**). Consistent with Pasam et al. (2012), these sub-populations of barley genotypes were clustered based on row types and geographic origin.

**Figure 2:**
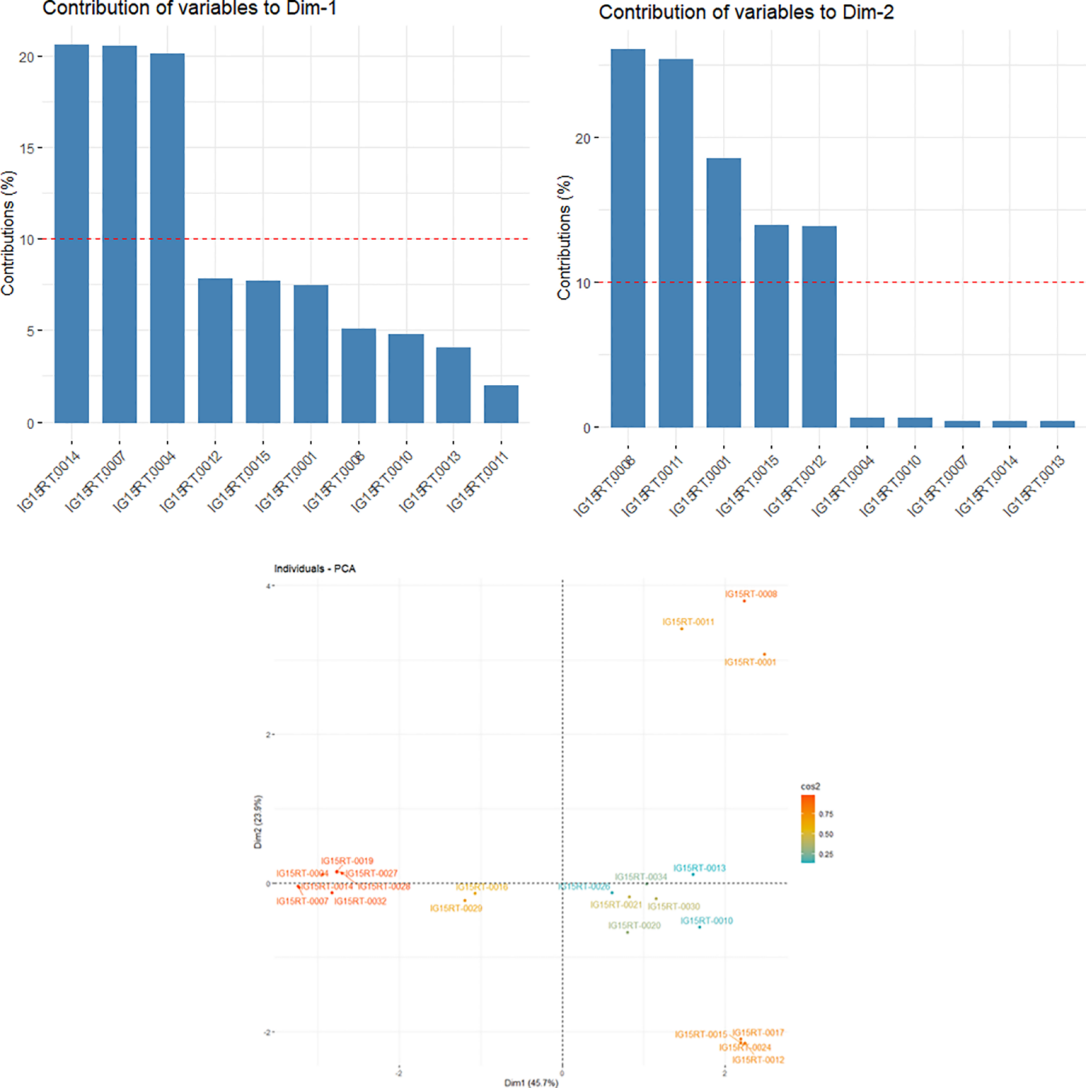
Estimated principal components (PCs) explaining the percentage of variation in 191 barley genotypes by utilizing GBS-SNP data.

A heatmap of the kinship matrix was computed using 21 062 SNPs to illustrate genetic relatedness among the barley genotypes in the panel, and a hierarchical tree was generated based on the kinship values (**Supplementary Figure 3**). Significant variation in correlation coefficients (from -0.16 to 1) between pairs of individual genotypes was observed in the kinship matrix. The genotypic groupings based on the population structure were also supported by the EMMA kinship algorithm. In the LD panel, the kinship values from EMMA matrix and eigenvalues from PCA were used to account for the relationships among the barley lines. The principal components (PC) were calculated from GBS-SNP markers data, with eigenvalues from 0.54 to 1.95. The principal component analysis (PCA) produced a set of three principal components, out of which PC1 and PC2 (with eigenvalues ≥1) accounted for a combined variance of 69.6% (**Figure 2**). Furthermore, the PC1 (x-axis) accounted for 45.7% of the explained variation, whereby the genotypes IG15RT-0004, IG15RT-0007, and IG15RT-0014 (InterGrain advanced breeding lines) exhibited a variation greater than 20%. Barley genotypes IG15RT-0001, IG15RT-0008, IG15RT-0011, IG15RT-0012, and IG15RT-0015, predominantly sourced from advanced breeding lines, accounted for a phenotypic variation exceeding 10%, with PC2 (y-axis) explaining 23.9% of the variation.

A phylogenetic analysis of 191 barley genotypes was carried out to evaluate whether the population structure could be inferred from the genome-wide genotypic data. Barley genotypes in the panel were clearly separated into different groups based on the Dice similarity coefficient values generated using the kinship matrix. A phylogenetic dendrogram was created using GBS-SNPs from the pairwise distances of 191 barley genotypes based on the root and shoot traits, which clustered all the genotypes into 10 phylogenetic groups (**Supplementary Figure 3**), with accession IG15RT_0147 being a single member of group 10. Compared with population structure grouping, the kinship matrix based on EMMA algorithm analysis (**Supplementary Figure 3**) clustered most genotypes in the groups 2 and 4 (containing 71 and 43 genotypes, respectively). Clustering groups 3 and 8 each comprised 10 barley genotypes, whereas groups 1 and 5 contained 16 and 20 genotypes, respectively. The remaining genotypes clustered into small groups 6 (3 genotypes), 7 (7), 9 (8), and 10 (1 genotype).

Generally, all barley lines with the same pedigree were clustered together as expected, but with a few exceptions. The lines IG15RT_0176 and IG15RT_0161 that had the Flinders/Fathom pedigree were clustered into groups 2 and 3, respectively (**Supplementary Figure 3**). All lines with the Hindmarsh/5/Hindmarsh/3/Hindmarsh/Scope//Hindmarsh/3/Hindmarsh pedigree were clustered into group 2, except line IG15RT_0011. The lines with the pedigree Vic9104/Dash/3/Skf/NsN//Onw/TR118/4/Vlamingh were clustered into two separate groups (IG15RT_0053 in group 1 and IG15RT_0114 in group 3). The lines with the pedigree Vlamingh/Hindmarsh were clustered into groups 2 (IG15RT_0139) and 4 (IG15RT_0142). Out of barley lines with the WABAR2312/WABAR2334 pedigree, the only line clustered separately (in group 3) was 1GRT_0024. Similarly, among the lines with the pedigree WABAR2534/Lockyer, only IG15RT_0193 was separated into group 2.

### 3.3 Linkage disequilibrium and genome-wide association analyses using root and shoot traits

**Figure 3:**
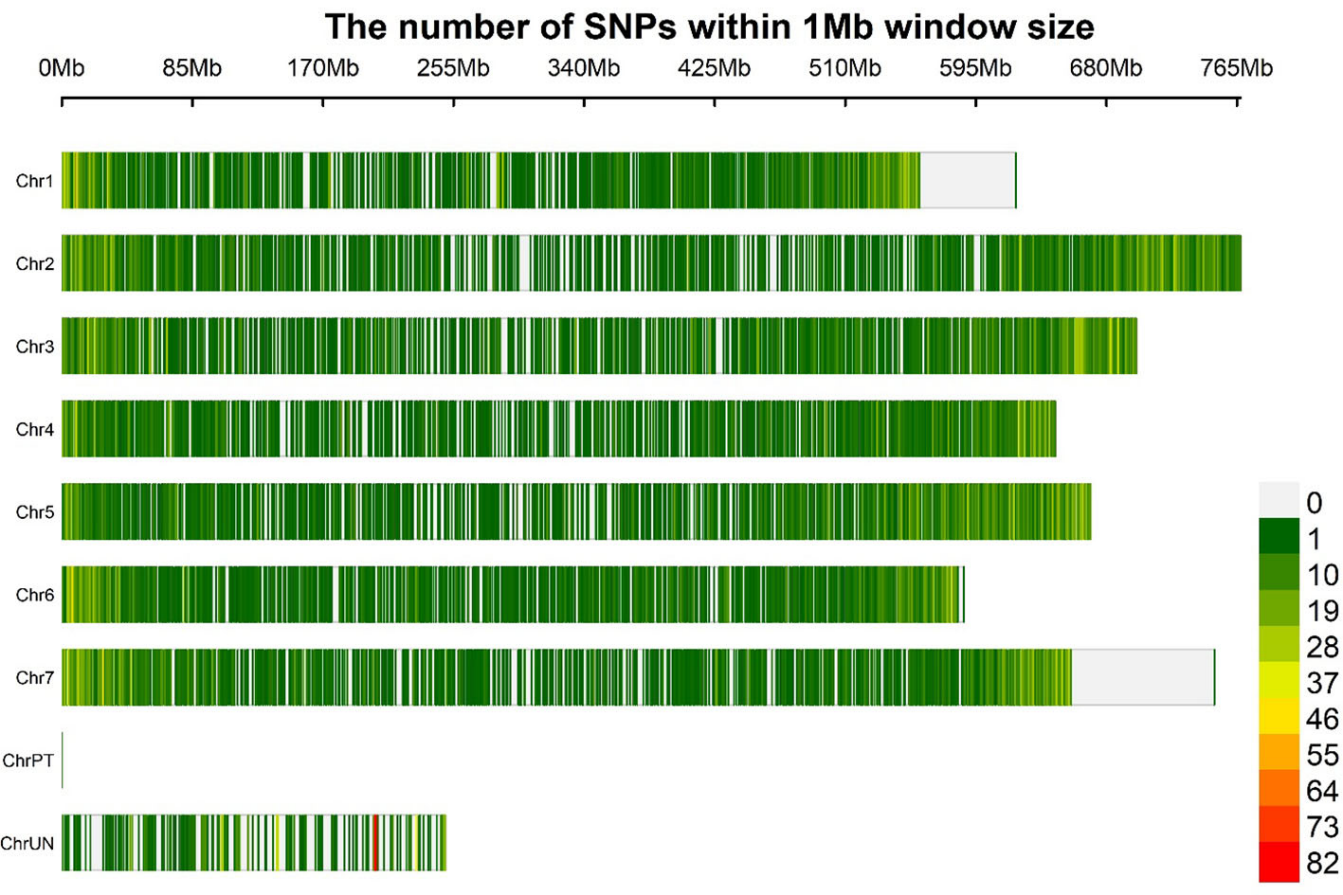
Distribution of SNP markers (produced after quality filtering) based on the physical map of the barley genome. The marker density is shown in the color legend on the right.

The LD decay was estimated using 21 062 GBS-SNPs to calculate pairwise distances (bp) among LD sites. A principal component was included in the GWAS for population based on the Bayesian information criterion (BIC) and maximum log likelihood values, which were applied in the model selection option in GAPIT using BLINK model. We used an r^2^ value rather than a normalized coefficient of LD (D′) because the r^2^ value is a more reliable parameter for comparing two alleles in a population (Slatkin, 2008). Moreover, the r^2^ values were negatively correlated with the physical distances (bp) between the loci. The intra-chromosomal LDs were calculated using 754 275 significant pairs of SNP comparisons that had physical distances ranging from 0.24 kb to 767.30 Mb across seven barley chromosomes (**Figure 3**). Significant LD was declared at *p<*0.001 and r^2^ ≥0.1. All the 191 barley lines exhibited a low LD decay when evaluated at a physical distance of 2.97 Mb. The LD decay estimation revealed a mean r^2^ value of 0.1 across the 191 barley lines, with a slightly higher r^2^ on chromosomes 2H, 5H, and 7H (**Supplementary Figure 4**). In the barley genome, 14 596 pairs of SNPs had high LD scores (r^2^ ≥0.8), most of which were on chromosome 3H. A strong LD was claimed at r^2^ >0.5 (Zhou et al., 2012). Regarding the LD decay on each chromosome, 3H had the slowest LD (4.32 Mb), followed by 2H (3.74 Mb). By contrast, chromosome 1H, with an average LD decay of 1.71 Mb, had the fastest LD decay. The LDs on the remaining chromosomes 4H, 5H, 6H, and 7H were 2.94, 2.68, 2.64, and 3.21 Mb, respectively.

The GWAS study employed the BLINK model to identify chromosome regions that exhibited significant MTAs for a total of 26 root and shoot traits. The statistical description of each studied trait was presented in **Supplementary Table 2**. A total of 1199 non-unique significant markers (*p ≤*0.001) were identified across all 26 root and shoot traits, with r^2^ values ranging from 0.08 to 0.41. The traits of topsoil root biomass (RB_top) and 20-100 cm deep root length (RL_lower) showed the strongest MTAs with r^2^ values of 0.41 and 0.36, respectively. The strongest MTA was observed for diameter of roots in 0-20 cm depth [RD_top, –log10 (p-value) 17.88] at the physical position of 634.09 Mb on chromosome 3H (**Table 2**). The status of significant MTAs was plotted against the threshold level of expected –log10 (p-value) as shown in the QQ-plots (**Supplementary Figure 5**). The highest number of significant MTAs (217 MTAs) was observed for diameter of roots in the 0-20 cm depth (RD_top), followed by root diameter (145 MTAs) in the 20-40 cm depth (RD_20), and length of thick roots (>0.25 mm diameter, i.e., diameter class length thick, DCL_thick) with 100 MTAs. By contrast, the lowest number of MTAs (15 MTAs) was observed in roots with diameter 0.075-0.25 mm (DCL_medium). In comparison, fewer significant MTAs were observed for shoot-related traits SH, Till, and SB (with 85, 56, and 26 MTAs, respectively). The genome-wide SNP markers that were most strongly (*p ≤*0.0001) associated with root and shoot traits are listed in **Table 2**.

The functional annotations for the sequences that carry most significant GBS-SNPs were identified by comparing physical locations (Mb) of the sequences that harbor SNPs with the annotated barley reference genome (Mascher et al., 2021). Proteins such as WRKY transcription factor protein family, glycogen synthase, auxin efflux carrier family protein, and thioredoxin superfamily protein were identified in these regions (**Table 2**), and they may play a role in barley root development (see also Abdel-Ghani et al., 2019).

### 3.4 Possible QTL regions for root and shoot traits

**Figure 4:**
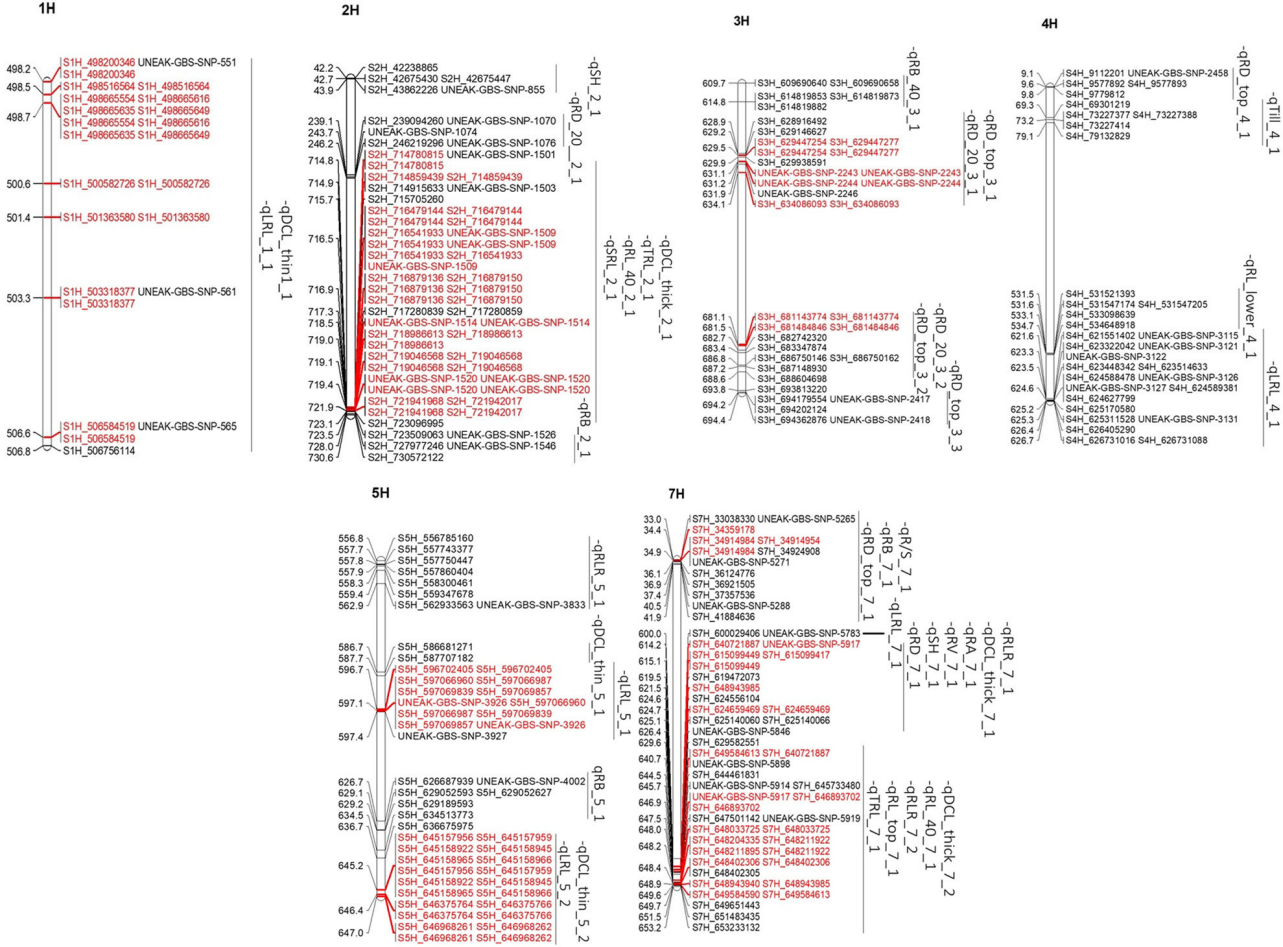
QTLs detected on six barley chromosomes. The markers highlighted in red are unique among QTLs on the same loci.

Multiple trait-associated markers in a short chromosome region may indicate that region as a possible QTL. All the root and shoot traits were analyzed to identify the putative QTL regions based on the physical chromosome positions of their trait-associated markers and the assumption that significant trait-associated SNPs within a 10-20 Mb physical interval in a chromosome (e.g. Jia et al., 2021) are linked to the same QTL (the genome-wide LD decay was 2.97 Mb). After determining physical locations of the 1199 significant trait-associated SNPs in the barley reference genome assembly (Barley Morex V3) (**Supplementary Table 3**), 271 highly significant MTAs were located in 40 chromosomal regions (QTLs), having five to 35 trait-associated SNPs in each QTL region. Out of these 40 QTL regions, 37 were associated with root traits, and only three were associated with shoot traits (**Figure 4**). The putative QTL regions associated with 19 traits (17 root and two shoot traits) were identified across six barley chromosomes (except 6H), having varying numbers of putative QTLs from two (1H) to 15 (7H). These QTLs had physical intervals from 0.53 to 10.2 Mb (**Figure 4**; **Table 3**).

**Table 3:** Significant quantitative trait loci (QTL) for 16 root traits and three shoot traits and their physical locations (Mb), number of significant trait-associated SNPs and proportion of phenotypic variation explained (PVE) by the QTL in the barley association panel.

Chromosome	QTL name	Traits ^a^	Starting physical location (Mb)	Interval distance (Mb)	Number of significant MTAs ^b^	PVE	Overlapping QTL ^c^
Chr 1H	qDCL_thin1_1	DCL_thin	498.20	8.56	14(10)	0.08-0.11	1
Chr 1H	qLRL_1_1	LRL	498.20	8.56	10(10)	0.08	1
Chr 2H	qSH_2_1	SH	42.24	2.87	10	0.06-0.08	
Chr 2H	qRD_20_2_1	RD_20	239.09	10.20	5	0.08-0.11	
Chr 2H	qDCL_thick_2_1	DCL_thick	710.96	10.20	24(11)	0.9-0.11	2
Chr 2H	qTRL_2_1	TRL	714.78	7.16	12(12)	0.07-0.09	2
Chr 2H	qRL_40_2_1	RL_40	714.92	10.20	20(8)	0.9-0.13	2
Chr 2H	qSRL_2_1	SRL	716.48	2.57	9(7)	0.06-0.08	2
Chr 2H	qRB_2_1	RB	725.79	4.78	6(5)	0.06-0.08	2
Chr 3H	qRB_40_3_1	RB_40	609.69	5.13	5	0.06-0.08	
Chr 3H	qRD_top_3_1	RD_top	628.92	5.17	9(5)	0.08-0.09	3
Chr 3H	qRD_20_3_1	RD_20	629.45	4.64	5(5)	0.06	3
Chr 3H	qRD_20_3_2	RD_20	681.14	6.01	5(2)	0.07-0.11	4
Chr 3H	qRD_top_3_2	RD_top	681.14	7.46	5(2)	0.07-0.11	4
Chr 3H	qRD_top_3_3	RD_top	693.81	0.53	6	0.1-0.11	
Chr 4H	qRD_top_4_1	RD_top	9.11	0.67	5	0.1-0.11	
Chr 4H	qTill_4_1	Till	69.3	9.83	5	0.08-0.11	
Chr 4H	qRL_lower_4_1	RL_lower	531.52	3.13	5	0.06-0.09	
Chr 4H	qLRL_4_1	LRL	621.55	5.18	18	0.06-0.08	
Chr 5H	qRLR_5_1	RLR	556.79	6.15	8	0.06-0.09	
Chr 5H	qDCL_thin_5_1	DCL_thin	586.68	10.20	8(6)	0.1-0.14	5
Chr 5H	qLRL_5_1	LRL	596.70	0.66	7(7)	0.08	5
Chr 5H	qRB_5_1	RB	626.69	9.99	7	0.08-0.13	
Chr 5H	qDCL_thin_5_2	DCL_thin	645.16	1.81	10(10)	0.06	6
Chr 5H	qLRL_5_2	LRL	645.16	1.81	10(10)	0.06	6
Chr 7H	qR/S_7_1	R/S	33.04	4.32	15(6)	0.07-0.14	7
Chr 7H	qRB_7_1	RB	34.36	0.56	6(6)	0.08	7
Chr 7H	qRD_top_7_1	RD_top	37.36	4.53	11	0.08-0.15	
Chr 7H	qLRL_7_1	LRL	600.03	4.20	5	0.06-0.11	
Chr 7H	qRLR_7_1	RLR	612.60	6.87	5	0.07-0.11	
Chr 7H	qDCL_thick_7_1	DCL_thick	614.23	10.20	5(5)		8
Chr 7H	qRA_7_1	RA	614.23	10.20	5(5)	0.07-0.11	8
Chr 7H	qRV_7_1	RV	614.23	10.20	5(5)	0.08-0.09	8
Chr 7H	qSH_7_1	SH	624.56	5.03	7	0.08-0.11	
Chr 7H	qRD_7_1	RD	625.14	1.28	6	0.06-0.08	
Chr 7H	qDCL_thick_7_2	DCL_thick	640.72	8.86	8(8)	0.08	9
Chr 7H	qRL_40_7_1	RL_40	640.72	7.68	10(9)	0.07-0.11	9
Chr 7H	qRLR_7_2	RLR	644.46	8.77	12	0.08-0.11	
Chr 7H	qRL_top_7_1	RL_top	645.73	2.67	5	0.09-0.11	
Chr 7H	qTRL_7_1	TRL	646.89	2.69	14(14)	0.07	9

^a.^ The trait abbreviations are listed in **Table 1**. The numbers in paratheses are total number of QTL for the trait identified in this study, if more than one.

^b.^ The numbers outside parentheses (if any) are the total numbers of trait-associated significant SNPs including these within 100 bp. The numbers in parenthesis are the numbers of the trait-associated significant SNPs that are at least 100 bp apart.

^c.^ The same numbers represent the QTL for different traits overlapping in the same genomic region.

The QTL regions were associated with 19 root and shoot traits, of which nine traits had only one QTL each; by contrast, root diameter of 0-20 cm deep (RD_top) and the longest root length (LRL) were each mapped to five QTL regions. Three putative QTLs were found for DCL_thick, DCL_thin, RB, RD_20, and RLR, whereas two QTLs were detected for RL_40, SH, and TRL (**Table 3**; **Supplementary Table 3**). Furthermore, nine QTLs were significant for at least two traits. The QTLs on 7H were significant for 13 traits, including root length, volume, diameter, biomass, and shoot height. These QTLs explained 6 to 15% of the phenotypic variation in different root and shoot traits (**Table 3**; **Supplementary Table 3**).

For a QTL region to be considered a hotspot due to the pleiotropic effects, at least two root and shoot traits were required to be linked to that particular region. We found nine hotspot QTLs in total, among which the 2H region from 714.78 to 727.98 Mb was important for five barley root traits (DCL_thick, TRL, RL_40, SRL, and RB) (**Table 3**; **Supplementary Table 3**). Out of the nine QTL regions that influenced multiple traits, the genomic region 640.72-651.91 Mb on 7H contained 49 MTAs for five root traits (*qDCL_thick_7_2, qRL_40_7_1, qRLR_7_2, qRL_top_7_1*, and *qTRL_7_1*) within the same region and about 440 annotated candidate genes. The remaining genomic regions on 1H (498.2-506.76 Mb), 3H (628.92-634.09 and 681.14-688.60 Mb), 5H (596.70-597.36 and 645.16-646.97 Mb), and 7H (34.36-34.92, 614.23-624.66, and 640.72-649.58 Mb) were associated with two to three traits each; therefore, these regions may be important for root architecture of barley.

## 4 Discussion

The root is the first plant organ to sense edaphic stress conditions, and thus plays a key role in plant responses to stress stimuli. An efficient phenotyping system has been developed to examine intrinsic genetic variation in barley root architecture for selecting superior root traits in breeding programs (Chen et al., 2011a; Chen et al., 2011b). In the current study, 13 out of 23 root architectural traits showed a variable growth response in the semi-hydroponic system based on CV ≥0.25 with the lowest *p*-value (<0.001) (Wang et al., 2021). The 13 root parameters represented the five major root traits (biomass, surface area, volume, length, and diameter). Previous studies indicated root diameter, length, and dry weight strongly influenced the physiological activities related to barley nutrient uptake (Heydari et al., 2018); the increased root area, volume, biomass, length, and diameter traits increased water uptake and improved stress tolerance (Manju et al., 2019). Furthermore, long roots can protect plants against stresses such as cold or drought (e.g. Hasanuzzaman et al., 2019; Oyiga et al., 2020); therefore, these five major root traits were considered the key root architectural traits in GWAS.

SNP markers generated by high-throughput genotyping technologies have been used for the genetic dissection of root architectural traits in many crops, such as maize (Sanchez et al., 2018; Zheng et al., 2020), wheat (Golan et al., 2018; Mehrabi et al., 2020; Salarpour et al., 2020), sorghum (Parra-Londono et al., 2018; Zheng et al., 2020), and rice (Li et al., 2017). They were also used to identify genetic regions controlling development of root traits in different barley genotypes (Arifuzzaman et al., 2014; Reinert et al., 2016; Xue et al., 2019; Oyiga et al., 2020). In the present study, 21 062 GBS-SNP markers were used to examine the barley association-mapping panel and identify MTAs for the 23 root and three shoot parameters. Among them, the significant GBS-SNPs were identified for 17 root and two shoot parameters (**Table 3**; **Supplementary Table 3**); most of these parameters were related to root length and biomass as well as shoot height, which agrees with the report by Mora et al. (2016).

The root system of a crop plant is quite complex (anatomically, physiologically, and genetically). To complement the increasing need for the knowledge on these aspects of the root system, the current study explored the population structure, genetic relatedness using complex clustering methods, and association of genomic regions with root-related traits in 191 barley genotypes. Previously, Munoz-Amatriaın et al. (2014) identified five subpopulations in 2417 accessions in the barley core collection using SNP markers. Fan et al. (2016) used 408 DArT markers to detect six subpopulations in 206 barley accessions in a QTL study of salinity stress. Hamblin et al. (2010) found seven distinct populations among 1816 barley accessions from the USA using 1416 SNP markers. Similarly, in the present study, the structure analysis separated the barley association panel into six subgroups (K=6) (**Figure 1**; **Supplementary Figure 2**), which is the same number as in the previous studies (e.g. Fan et al., 2016). Multiple subgroups identified in this panel are not unexpected because the panel consists of barley accessions with diverse origins. The most accessions in the panel are semi-advanced and parental materials from the InterGrain Pty Ltd breeding program that breeds barley cultivars adapted to diverse environments throughout Australia. However, the parental accessions in the panel included also advanced breeding lines from the former Department of Agriculture and Food program in Queensland (Australia) and commercial varieties from the rest of Australia, as well as varieties and advanced breeding lines from Europe and Ethiopia.

In the current study, the methods such as population structure, kinship and clustering analyses were employed to get insight into the stratification of the barley collection. We corrected for population structure to minimize residual inflation of the test statistics due to unidentified population structure effects (cf. Cockram et al., 2008). Based on the phylogenetic relationships determined by the kinship matrix, the barley panel used in the present study formed two main clades with 10 different subgroups (**Supplementary Figure 3**). All the genotypes were separated into multi-line clusters based on their origins and breeding history, with the exception of IG15RT_0147 (an Ethiopian landrace), which is consistent with prior research (Igartua et al., 2013). Hence, the dendrogram clades were a good representation of genetic relatedness among the barley lines based on the 17 selected root traits. In comparison, Dai et al. (2012) grouped a set of 185 Tibetan barley accessions into two main clades and eight subclasses using 1307 DArTs, whereas Close et al. (2009) identified six distinct UPGMA groups from 37 barley accessions using 1301 SNPs. The differences in UPGMA clustering may be due to the different sources of plant populations and the marker systems used in different studies.

Previous studies have demonstrated the influence of a statistical model used on the significance of MTAs (Comadran et al., 2011; Pasam et al., 2012). By updating the statistical techniques, false positives may be reduced. The mixed linear model (MLM) includes population structure and kinship that may inflate *p* values in GWAS, and may also eliminate signals from the known genes that are present as background noise (Yu et al., 2006). Hence, a new statistical method (BLINK) was developed, combining fixed effect model (FEM), Bayesian information criterion, and linkage disequilibrium information to solve the problem of testing multiple loci in MLM. We used BLINK in the present study. The BLINK model accounts for heterogeneity of genetic background and decreases false positive associations (Yu et al., 2006). In our study, the FDR cut-off value used was 0.05, which is stringent and expected to get false positive results in only 5% of analyses, making it more reliable than the cut-off value of 0.1.

LD decay information is a key factor for determining the minimum density of markers required in association mapping and marker-assisted selection. Our LD analysis identified the most responsive genomic regions harboring significant MTAs for root traits (i.e. RA, RB, RD, TRL, and RV), with the most significant MTAs for root diameter (RD) and total root length (TRL). Our LD analysis identified 2.97 Mb as the average LD decay length, which is slightly shorter than 3.5 Mb using 350 barley accessions reported by Mwando et al. (2020). In comparison, Karunarathne et al. (2020) observed slower LD decay (10.6 Mb) than in the present study by employing 282 barley accessions. Furthermore, Comadran et al. (2011) examined the whole-genome pattern with a fast decay of LD in 190 elite barley germplasm (North and West European and North American) by using 4596 SNP markers, out of which 2132 had minor allele frequencies higher than 0.1 in association mapping, and 91.2% of these 2132 SNPs were mapped within 10 cM of their original genetic map positions, confirming the power of GWAS. In another study, Reinert et al. (2016) employed 5892 SNP markers with the proportion of the phenotypic variation explained (marker r^2^) decreasing from 0.17 to<0.1 in chromosome 7H and being<0.1 for chromosomes 1H to 6H. In comparison, we report here the average r^2^ of 0.1 across all strongly associated LD sites (**Supplementary Figure 4**), indicating that GBS-SNPs used in the current study were sufficiently robust for identifying significant MTAs (cf. Zhou, 2011; Naz et al., 2014; Mahalingam et al., 2020). Cai et al. (2013) found that the r^2^ value ≥0.1 in LD decay may provide a significant output and have a large effect on barley root traits in GWAS. In the current study, we found the high LD extending along each chromosomal position because the mixed model significantly reduced the long range and background LD, which might happen due to the intra- and inter-chromosomal SNP-SNP interactions (Close et al., 2009). Furthermore, the marker pairs with strong LD patterns over long distances observed in the current study may be useful for understanding the genotypic selection, recombination and their breeding history (cf. Munoz-Amatriaın et al., 2014), reflecting the non-static nature of LD that is influenced not only by physical or genetic distances, but also by various other factors such as genetic admixture (McVean, 2006; Comadran et al., 2011).

**Figure 5:**
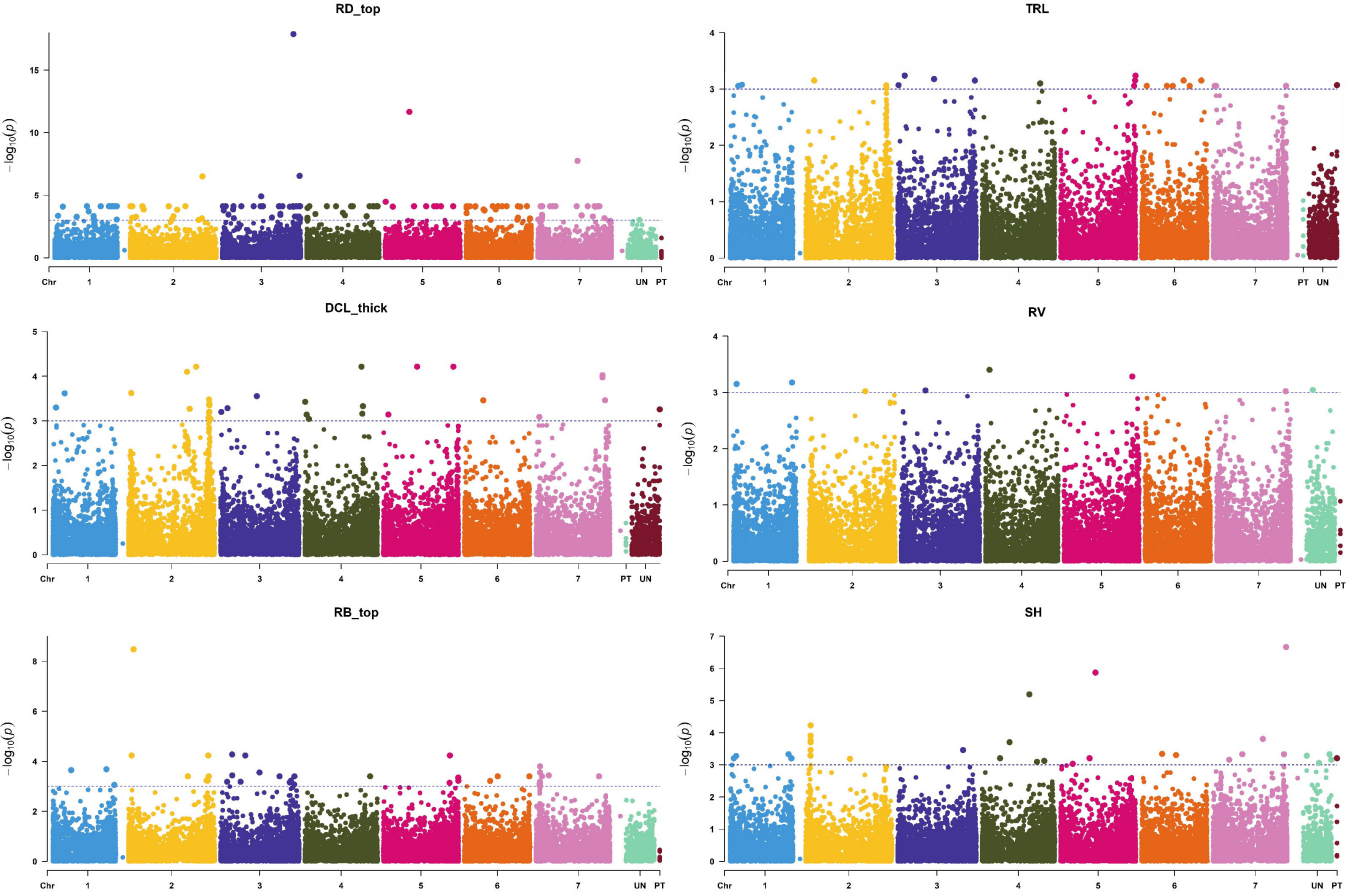
Manhattan plots of root [surface area (RA), biomass (RB), volume (RV), average diameter (RD), and total length (TRL)] and shoot traits [shoot height (SH)] with the strongest marker-trait association. The X-axis is the genomic position of the SNPs in bp, and the Y-axis is the –log10(*p*-values).

In the current study, the most significant MTAs were found for root length, surface area, biomass, diameter, and volume (**Figure 5**), which was in agreement with the published reports (Comadran et al., 2011; George et al., 2014). Wu et al. (2015) found the strongest MTA value for barley root length on chromosome 2H, which was only slightly lower than our value for total root length (TRL, **Figure 5**). In another study, Reinert et al. (2016) found strong MTAs for root length on chromosome 5H (genetic position of 93.20 cM) using the same threshold level of *p*-value (0.001) as we used in the present study. The physical to genetic distance on barley chromosomes was 1-10 Mb per cM in distal regions, but it could be 100-500 Mb size in the pericentromeric region (Ariyadasa et al., 2014). In our study, some of the identified QTL regions were mapped in a larger marker interval; it was due to low coverage of SNP markers in those specific regions.

We identified 40 QTL regions within 271 MTAs (**Supplementary Table 3**) for different root and shoot traits with seven genomic regions influencing multiple root or shoot traits in the barley association panel. Most of these MTAs were co-localized at the putative QTL regions on chromosomes 2H, 5H, and 7H. Similarly, Robinson et al. (2016) identified QTL on chromosome 5H for root number in barley, which was similar to the genomic region we detected for root length traits. Arifuzzaman et al. (2014) detected three novel QTLs (*QRl.S42.2H, QRl.S42.3 H*, and *QRl.S42.5H*) for root length in barley on 2H (41.1 cM), 3H (118.72 cM), and 5H (125.1 cM) linkage groups using microsatellite (SSR) and DArT markers; these QTL regions were also significant for the root length and the related traits found in the present study (**Supplementary Table 3**). Reinert et al. (2016) detected 17 putative QTLs related to five key root and shoot traits of barley. Jia et al. (2019) identified 65 MTAs for root architectural traits, mainly on chromosomes 2H and 6H, using 221 spring barley lines and the significant threshold *p*-value of 0.001. The significant QTLs in our study, specifically *qDCL_thick_2_1, qTRL_2_1, qRL_40_2_1, qSRL_2_1*, and *qRB_2_1* on 2H (**Supplementary Table 3**), were also detected by Jia et al. (2019) as QTLs related to root length and biomass (*qRSD3* at 56.52 cM and *qTSRL3* at 76.2 cM). Similarly, the QTL regions (*qRD_top_3_1* and *qRD_20_3_1*) identified on 3H in the current study (**Supplementary Table 3**) were the same as *qRSA6* at 67.92 cM in Jia et al. (2019), highlighting the importance of these genomic regions in governing root morphology. Previous studies identified QTLs associated with shoot height and biomass and root dry weight in barley genome (Arifuzzaman et al., 2014; Naz et al., 2014). QTLs for root biomass were found on chromosomes 1H, 2H, and 5H. The QTL regions for root length were discovered on chromosomes 1H, 2H, 3H, 4H, 5H, and 7H (Arifuzzaman et al., 2014; Naz et al., 2014; Reinert et al., 2016). Importantly, QTLs for root surface area, root volume, and root diameter we found in the present study have not been reported to date.

In the present study, the chromosome region of 498.20-506.76 Mb on 1H that had 10 MTAs corresponded to a major QTL for root traits (i.e., DCL_thin and LRL). On 2H, a QTL hotspot region for root traits such as DCL_thick, TRL, RL_40, SRL, and RB comprised 44 MTAs at the physical interval of 714.78-727.98 Mb (**Table 3**; **Supplementary Table 3**). In addition, two QTL hotspot regions linked with root diameter (RD_top and RD_20) traits were found at the intervals 628.92-634.09 Mb on 3H (containing five significant MTAs) and 681.14-687.15 Mb (with two MTAs). Similarly, two major regions were identified at 586.68-597.65 Mb and 645.16-646.97 Mb on 5H chromosome containing seven and 10 significant MTAs, respectively. Three major QTL regions were located in three intervals on 7H chromosome with six MTAs each located at 33.04-37.36 Mb and 614.23-624.66 Mb and 14 MTAs at 640.72-649.58 Mb for multiple root traits (DCL_thick, RL_40, RA, RB, R/S, RV, and TRL). These identified regions explained 6 to 15% of the phenotypic variation (**Table 3**). The QTLs for two root parameters (*qTRL_2_1* and *qDCL_thick_2_1*) were localized in the same region on chromosome 2H, a region similar to the previously reported QTL for root length (Arifuzzaman et al., 2014) and lateral root growth (Guo et al., 2018).

Among the QTLs identified in this study, the QTL region 640.72-653.23 Mb on 7H was significant for five root traits (DCL_thick, RL_40, RLR, RL_top, and TRL); thus, this region may harbor multiple tightly linked QTLs or a pleiotropic QTL; therefore, this is an important QTL for breeding programs to improve barley root architecture (**Figure 5**). This QTL region contains more than 400 annotated high-confidence genes. If the overlapping of QTLs for various traits was due to a pleiotropic effect, only one or a few of these genes may be the candidate causal gene(s) for the QTL responsible for the development of barley root architecture traits; thus, further fine mapping of the QTL will narrow down the QTL region and shorten the candidate list to determine the final causal gene(s) for the QTL. It is tempting to speculate that candidate genes identified in that particular region of 7H (i.e. HORVU7Hr1G025180 and HORVU7Hr1G009640) with functional annotation of glyceraldehyde-3-phosphate dehydrogenase C2 and auxin response factor 19, respectively (Abdel-Ghani et al., 2019), may be influencing barley root architecture. Another important QTL with 62 MTAs associated with five different parameters (DCL_thick, TRL, RL_40, SRL and RB) was located in the 13.2 Mb interval (714.78-727.98 Mb) on chromosome 2H; this QTL may be important for root architecture and can be used in breeding for root system improvement.

## 5 Conclusions

We identified 1199 significant MTAs for 17 root and two shoot traits in barley germplasm. The 37 significant QTLs for these traits were detected in the association-mapping panel, with three QTLs for the shoot and 34 for the root traits. Root diameter and length had overlapped with the QTLs for root surface area and volume, and thus appeared to be the most promising root traits for marker-assisted selection and breeding deep-rooting barley varieties for environmental adaptation (especially in drying soils). The putative QTLs for the important root traits and their associated markers may be useful genomic resources for marker-assisted selection to introgress these root trait QTLs into new cultivars to improve their adaptation to specific environments. Further validation of the significant markers should rely on larger populations to increase the power of genetic association studies.

## Data availability statement

The datasets presented in this study can be found in online repositories. The names of the repository/repositories and accession number(s) can be found below: BioProject: PRJNA1017445.

## Author contributions

MF: Formal Analysis, Methodology, Writing – original draft. DM: Data curation, Supervision, Writing – review & editing. GB: Data curation, Writing – review & editing, Methodology, Validation. AB: Data curation, Methodology, Writing – review & editing. PS: Data curation, Methodology, Writing – review & editing. AD: Writing – review & editing, Conceptualization. ZR: Conceptualization, Writing – review & editing, Project administration, Supervision.

## Supplementary material

Supplementary Figure 1 Heterozygosity among 191 genotypes and GBS-SNPs used in the current study. The values on the X-axis indicate heterozygous allele proportions ranging from 0 to 1 in the association mapping panel.

Supplementary Figure 2 The barley association panel population was separated into six subpopulations (K=6) based on SNP markers (top graph) along with principal component (PC) plot of first three PCs (bottom graph). In the top graph, each color in population structure represents a different subpopulation. Numbers on the Y-axis represent subpopulation membership values.

Supplementary Figure 3
**A** heatmap and dendrogram of a kinship matrix based on the Efficient Mixed-Model Association (EMMA) algorithm using GBS-SNP markers in the barley association panel.

Supplementary Figure 4 Linkage disequilibrium (LD) decay over physical distances (bp) for the whole genome and all seven chromosomes individually. The green line represents a half-decay distribution. The LD was computed on sliding windows of adjacent markers where each symbol represented a distance between paired loci against their squared correlation coefficient. Pair-wise LD values of polymorphic sites were calculated from a rapid 1000 shuffle permutation test. Each symbol indicates the squared correlation coefficient of a pair of distances between two markers on the window.

Supplementary Figure 5 Quantile-quantile (Q-Q) plots for strongly associated key root [biomass (RB), surface area (RA), volume (RV), average diameter (RD) and total root length (TRL)] and shoot traits [height (SH)]. The Y-axis is the observed –log10(p-value), and the X-axis is the expected –log10(*p*-value) under the assumption that the *p*-values follow a uniform [0,1] distribution.
