# Supplementary material for: Genetic characterization of root architectural traits in barley (Hordeum vulgare L.) using SNP markers
Source: Front Plant Sci. 2023 Oct 4;14:1265925. doi: 10.3389/fpls.2023.1265925 (PMC10582755; doi:10.3389/fpls.2023.1265925)
Supplement: Supplementary file 1 [file Table_1.docx]

**Supplementary Table S1:** List of barley genotypes phenotyped in this study, including breeding lines and some parents used in InterGrain barley (IGB) breeding program. The grouping is based on the population structure (K=6).

| **Group #** | **Code** | **Name** | **Group #** | **Code** | **Name** | **Group #** | **Code** | **Name** | **Group #** | **Code** | **Name** | **Group #** | **Code** | **Name** |
| --- | --- | --- | --- | --- | --- | --- | --- | --- | --- | --- | --- | --- | --- | --- |
| G1 | IG15RT_0001 | IGB1320 | G3 | IG15RT_0060 | IGB1323T | G2 | IG15RT_0119 | IGB1424T | G3 | IG15RT_0163 | IGB1515 | G4 | IG15RT_0205 | IGB1574 |
| G1 | IG15RT_0004 | IGB1335T | G2 | IG15RT_0061 | IGB1333T | G2 | IG15RT_0120 | IGB1336T | G2 | IG15RT_0164 | IGB1516 | G4 | IG15RT_0206 | IGB1575 |
| G2 | IG15RT_0007 | IGB1238 | G2 | IG15RT_0062 | IGB1422T | G1 | IG15RT_0121 | IGB1319 | G5 | IG15RT_0165 | IGB1517 | G4 | IG15RT_0207 | Lx09.76-022 |
| G1 | IG15RT_0008 | Vlamingh | G2 | IG15RT_0063 | IGB1419T | G5 | IG15RT_0122 | IGB1330T | G3 | IG15RT_0166 | IGB1518 | G4 | IG15RT_0208 | Lx09.83-019 |
| G6 | IG15RT_0010 | Shepherd | G1 | IG15RT_0064 | IGB1412 | G6 | IG15RT_0123 | Propino | G5 | IG15RT_0167 | IGB1519 | G4 | IG15RT_0209 | Lx09.98-003 |
| G1 | IG15RT_0011 | IGB1339T | G1 | IG15RT_0067 | IGB1425T | G2 | IG15RT_0126 | IGB1334T | G6 | IG15RT_0168 | IGB1520 | G4 | IG15RT_0210 | NRB120142-007 |
| G6 | IG15RT_0012 | IGB1308 | G2 | IG15RT_0069 | IGB1416T | G4 | IG15RT_0127 | 06S334-29 | G5 | IG15RT_0169 | IGB1521 | G4 | IG15RT_0211 | NRB120444-007 |
| G6 | IG15RT_0013 | IGB1217 | G4 | IG15RT_0071 | IGB1322T | G4 | IG15RT_0128 | 07S462-65 | G6 | IG15RT_0170 | IGB1522 | G4 | IG15RT_0212 | NRB120562 |
| G2 | IG15RT_0014 | IGB1239 | G4 | IG15RT_0072 | IGB1315 | G4 | IG15RT_0129 | 07S539-39 | G1 | IG15RT_0171 | IGB1523 | G4 | IG15RT_0213 | NRB120579-008 |
| G5 | IG15RT_0015 | IGB1210T | G4 | IG15RT_0074 | Quench | G4 | IG15RT_0130 | 07S543-106 | G1 | IG15RT_0172 | IGB1524 | G4 | IG15RT_0214 | NRB120766-006 |
| G3 | IG15RT_0016 | IGB1324T | G4 | IG15RT_0075 | IGB1303 | G5 | IG15RT_0131 | 08S846-016 | G1 | IG15RT_0173 | IGB1525 | G4 | IG15RT_0215 | NRB120772-012 |
| G6 | IG15RT_0017 | IGB1310 | G2 | IG15RT_0076 | IGB1418T | G6 | IG15RT_0132 | 09S526-119 | G5 | IG15RT_0174 | IGB1526 | G4 | IG15RT_0216 | NRB120917-008 |
| G2 | IG15RT_0019 | IGB1217 | G6 | IG15RT_0078 | Gairdner | G5 | IG15RT_0133 | 10M73SD-061 | G6 | IG15RT_0175 | IGB1527 | G4 | IG15RT_0217 | Sloop_VIC |
| G4 | IG15RT_0020 | Publican | G6 | IG15RT_0080 | IGB1321T | G5 | IG15RT_0134 | 10S692N-11 | G5 | IG15RT_0176 | IGB1528 | G4 | IG15RT_0218 | Dragon |
| G4 | IG15RT_0021 | Wimmera | G6 | IG15RT_0082 | Buloke | G5 | IG15RT_0135 | 10S692N-157 | G4 | IG15RT_0177 | IGB1529 | G4 | IG15RT_0219 | Sirish |
| G4 | IG15RT_0024 | IGB1307 | G6 | IG15RT_0083 | IGB1456 | G6 | IG15RT_0136 | 10S692N-236 | G4 | IG15RT_0178 | IGB1530 | G4 | IG15RT_0220 | IGB1533 |
| G4 | IG15RT_0026 | Funders | G2 | IG15RT_0087 | IGB1428T | G6 | IG15RT_0137 | 10S770D-149 | G4 | IG15RT_0179 | IGB1531 | G4 | IG15RT_0221 | IGB1534 |
| G1 | IG15RT_0027 | IGB1427T | G4 | IG15RT_0088 | Scope | G1 | IG15RT_0138 | 10S881N-107 | G4 | IG15RT_0180 | IGB1532 | G6 | IG15RT_0222 | IGB1535 |
| G2 | IG15RT_0028 | IGB1426T | G4 | IG15RT_0089 | IGB1404 | G3 | IG15RT_0139 | 10S881N-141 | G4 | IG15RT_0181 | IGB1550 | G6 | IG15RT_0223 | Laureate |
| G3 | IG15RT_0029 | IGB1327T | G4 | IG15RT_0090 | IGB1311 | G1 | IG15RT_0140 | 10S881N-298 | G4 | IG15RT_0182 | IGB1551 | G6 | IG15RT_0224 | IGB1536 |
| G6 | IG15RT_0030 | IGB1305 | G1 | IG15RT_0091 | IGB1317 | G1 | IG15RT_0141 | 10S881N-40S | G4 | IG15RT_0183 | IGB1552 | G6 | IG15RT_0225 | IGB1537 |
| G2 | IG15RT_0032 | Latrobe | G4 | IG15RT_0092 | IGB1331 | G1 | IG15RT_0142 | 10S881N-421 | G4 | IG15RT_0184 | IGB1553 | G4 | IG15RT_0226 | VB0611 |
| G4 | IG15RT_0034 | IGB1304 | G4 | IG15RT_0093 | IGB1316 | G5 | IG15RT_0143 | 10S881N-421 | G4 | IG15RT_0185 | IGB1554 | G4 | IG15RT_0227 | VB0916 |
| G4 | IG15RT_0036 | Grout | G1 | IG15RT_0094 | IGB1429T | G4 | IG15RT_0144 | 11M014-013-011 | G4 | IG15RT_0186 | IGB1555 |  |  |  |
| G5 | IG15RT_0037 | Fathom | G6 | IG15RT_0096 | IGB1467 | G4 | IG15RT_0145 | 11M048HT-019F4 | G4 | IG15RT_0187 | IGB1556 |  |  |  |
| G2 | IG15RT_0038 | IGB1431T | G6 | IG15RT_0097 | IGB1235 | G4 | IG15RT_0146 | Compass | G4 | IG15RT_0188 | IGB1557 |  |  |  |
| G4 | IG15RT_0039 | Mundah | G6 | IG15RT_0100 | IGB1406 | G6 | IG15RT_0147 | ETH295B | G3 | IG15RT_0189 | IGB1558 |  |  |  |
| G4 | IG15RT_0040 | IGB1211 | G2 | IG15RT_0101 | IGB1414T | G4 | IG15RT_0148 | Garner | G6 | IG15RT_0190 | IGB1559 |  |  |  |
| G1 | IG15RT_0041 | IGB1432T | G2 | IG15RT_0102 | IGB1413T | G4 | IG15RT_0149 | IGB1501 | G6 | IG15RT_0191 | IGB1560 |  |  |  |
| G3 | IG15RT_0042 | IGB1329T | G6 | IG15RT_0103 | IGB1301 | G4 | IG15RT_0150 | IGB1502 | G1 | IG15RT_0192 | IGB1561 |  |  |  |
| G5 | IG15RT_0043 | IGB1469 | G6 | IG15RT_0105 | IGB1463 | G4 | IG15RT_0151 | IGB1503 | G5 | IG15RT_0193 | IGB1562 |  |  |  |
| G2 | IG15RT_0044 | IGB1240 | G5 | IG15RT_0106 | IGB1212T | G4 | IG15RT_0152 | IGB1504 | G6 | IG15RT_0194 | IGB1563 |  |  |  |
| G4 | IG15RT_0045 | Baudin | G5 | IG15RT_0108 | IGB1312 | G4 | IG15RT_0153 | IGB1505 | G6 | IG15RT_0195 | IGB1564 |  |  |  |
| G4 | IG15RT_0047 | Forensic | G6 | IG15RT_0109 | IGB1302 | G4 | IG15RT_0154 | IGB1506 | G6 | IG15RT_0196 | IGB1565 |  |  |  |
| G2 | IG15RT_0048 | IGB1338T | G3 | IG15RT_0111 | IGB1326T | G4 | IG15RT_0155 | IGB1507 | G6 | IG15RT_0197 | IGB1566 |  |  |  |
| G6 | IG15RT_0052 | Henley | G2 | IG15RT_0112 | IGB1337T | G2 | IG15RT_0156 | IGB1508 | G4 | IG15RT_0198 | IGB1567 |  |  |  |
| G1 | IG15RT_0053 | IGB1140 | G4 | IG15RT_0113 | Commander | G2 | IG15RT_0157 | IGB1509 | G4 | IG15RT_0199 | IGB1568 |  |  |  |
| G2 | IG15RT_0054 | IGB1418T | G4 | IG15RT_0114 | IGB1139 | G5 | IG15RT_0158 | IGB1510 | G4 | IG15RT_0200 | IGB1569 |  |  |  |
| G4 | IG15RT_0056 | Skipper | G3 | IG15RT_0115 | IGB1328T | G5 | IG15RT_0159 | IGB1511 | G4 | IG15RT_0201 | IGB1570 |  |  |  |
| G4 | IG15RT_0057 | Oxford | G3 | IG15RT_0116 | IGB1325T | G6 | IG15RT_0160 | IGB1512 | G6 | IG15RT_0202 | IGB1571 |  |  |  |
| G4 | IG15RT_0058 | IGB1309 | G6 | IG15RT_0117 | Bass | G6 | IG15RT_0161 | IGB1513 | G6 | IG15RT_0203 | IGB1572 |  |  |  |
| G1 | IG15RT_0059 | IGB1332 | G3 | IG15RT_0118 | IGB1214T | G3 | IG15RT_0162 | IGB1514 | G6 | IG15RT_0204 | IGB1573 |  |  |  |
