# Supplementary material for: Genetic characterization of root architectural traits in barley (Hordeum vulgare L.) using SNP markers
Source: Front Plant Sci. 2023 Oct 4;14:1265925. doi: 10.3389/fpls.2023.1265925 (PMC10582755; doi:10.3389/fpls.2023.1265925)
Supplement: Supplementary file 2 [file Table_2.docx]

**Supplementary Table S2** Statistical test values for individual traits based on the BLINK model

| **Trait** | ***p*-value** | **MAF** | **r^2^ of model without SNP** | **r^2^ of model with SNP** | **FDR Adjusted *p*-value** | **Effect** | **Genetic var** | **Residual var** | **-2LnLikelihood** |
| --- | --- | --- | --- | --- | --- | --- | --- | --- | --- |
| RA | 0.00092 | 0.18 | 0.14 | 0.19 | 0.90 | 6.38 | 2,121 | 1,206 | 1,907 |
| RB | 0.00111 | 0.22 | 0.29 | 0.32 | 0.42 | -28.33 | 40,210 | 68,330 | 2,594 |
| RB_20 | 0.00080 | 0.30 | 0.19 | 0.22 | 0.59 | 3.56 | 6,559 | 5,656 | 2,184 |
| RB_40 | 0.00115 | 0.15 | 0.11 | 0.15 | 0.61 | -21.91 | 13,684 | 14,401 | 2,347 |
| LRN | 0.00132 | 0.08 | 0.22 | 0.25 | 0.77 | 0.68 | 0.35 | 0.76 | 506 |
| R/S | 0.00103 | 0.18 | 0.27 | 0.30 | 0.42 | 10.15 | 941 | 1,826 | 1,908 |
| RB_top | 0.00112 | 0.13 | 0.39 | 0.41 | 0.62 | -5.64 | 11,385 | 13,086 | 2,310 |
| RD | 0.00079 | 0.21 | 0.13 | 0.16 | 0.66 | 0.00 | 0 | 0 | -648 |
| TRL | 0.00117 | 0.20 | 0.12 | 0.17 | 0.57 | -145.26 | 228,412 | 235,936 | 2,844 |
| RL_20 | 0.00122 | 0.17 | 0.02 | 0.08 | 0.96 | 35.34 | 47,795 | 42,867 | 2,542 |
| RL_40 | 0.00109 | 0.31 | 0.05 | 0.10 | 0.50 | -19.28 | 16,927 | 41,092 | 2,494 |
| DCL_med | 0.00110 | 0.14 | 0.09 | 0.15 | 0.94 | 32.49 | 11,341 | 8,047 | 2,257 |
| DCL_thick | 0.00099 | 0.26 | 0.27 | 0.31 | 0.37 | 7.98 | 53,498 | 71,384 | 2,591 |
| RL_lower | 0.00141 | 0.16 | 0.33 | 0.36 | 0.83 | 10.93 | 0.45 | 0.8 | 531 |
| RLR | 0.00090 | 0.20 | 0.09 | 0.13 | 0.45 | -0.06 | 0.07 | 0.04 | -16.54 |
| SRL | 0.00097 | 0.32 | 0.12 | 0.17 | 0.96 | 7.22 | 1,081 | 1,956 | 1,944 |
| DCL_thin | 0.00069 | 0.25 | 0.03 | 0.08 | 0.38 | -2.25 | 45,398 | 38,479 | 2,541 |
| LRL | 0.00086 | 0.19 | 0.04 | 0.09 | 0.34 | -7.20 | 0.74 | 0.83 | 548 |
| RL_top | 0.00012 | 0.22 | 0.19 | 0.23 | 0.11 | -55.47 | 47,283 | 60,729 | 2,584 |
| RV | 0.00121 | 0.16 | 0.14 | 0.19 | 0.98 | 0.09 | 0.09 | 0.05 | 71.64 |
| SB | 0.00129 | 0.15 | 0.14 | 0.15 | 0.86 | -0.01 | 192,040 | 179,791 | 2,800 |
| RD_top | 0.00065 | 0.09 | 0.12 | 0.16 | 0.09 | 0.00 | 0.46 | 0.7 | 479 |
| RD_20 | 0.00081 | 0.06 | 0.05 | 0.09 | 0.16 | -0.01 | 1,012 | 1,428 | 1,891 |
| SH | 0.00095 | 0.21 | 0.30 | 0.33 | 0.40 | 0.10 | 6.28 | 5.05 | 918 |
| RD_40 | 0.00009 | 0.06 | 0.08 | 0.14 | 0.04 | 0.01 | 886 | 1,747 | 1,904 |
| Till | 0.00120 | 0.11 | 0.12 | 0.17 | 0.71 | -0.03 | 0.12 | 0.20 | 268 |

For trait codes, see Table 1.
