# Supplementary material for: Genetic characterization of root architectural traits in barley (Hordeum vulgare L.) using SNP markers
Source: Front Plant Sci. 2023 Oct 4;14:1265925. doi: 10.3389/fpls.2023.1265925 (PMC10582755; doi:10.3389/fpls.2023.1265925)
Supplement: Supplementary file 3 [file Table_3.docx]

**Supplementary Table S3** Significant Quantitative Trait Loci (QTL) for 16 root traits and three shoot traits and their linked markers, physical locations (Mb) and r^2^ values in the barley association panel.

| **QTL Name** | **Trait*** | **Marker**** | **Alleles** | **Linkage Group** | **Physical location (Mb) ***** | **Marker r^2^** | **-log 10 (*p*-value)** |
| --- | --- | --- | --- | --- | --- | --- | --- |
| qDCL_thin1_1 | DCL_thin | S1H_498200346 | G/A | 1H | 498.20 | 0.08 | 3.52 |
|  |  | UNEAK-GBS-SNP-551 | G/A | 1H | 498.20 | 0.06 | 2.84 |
|  |  | S1H_498516564 | C/A | 1H | 498.52 | 0.09 | 4.19 |
|  |  | S1H_498665554 | C/T | 1H | 498.67 | 0.08 | 4.10 |
|  |  | S1H_498665616 | T/C | 1H | 498.67 | 0.07 | 3.53 |
|  |  | S1H_498665635 | T/C | 1H | 498.67 | 0.07 | 3.53 |
|  |  | S1H_498665649 | G/A | 1H | 498.67 | 0.09 | 4.34 |
|  |  | S1H_500582726 | C/T | 1H | 500.58 | 0.06 | 2.99 |
|  |  | S1H_501363580 | T/C | 1H | 501.36 | 0.06 | 3.01 |
|  |  | S1H_503318377 | T/C | 1H | 503.32 | 0.07 | 3.12 |
|  |  | UNEAK-GBS-SNP-561 | G/T | 1H | 503.32 | 0.07 | 2.87 |
|  |  | S1H_506584519 | T/A | 1H | 506.58 | 0.06 | 3.04 |
|  |  | UNEAK-GBS-SNP-565 | T/A | 1H | 506.58 | 0.06 | 2.91 |
|  |  | S1H_506756114 | T/C | 1H | 506.76 | 0.06 | 2.81 |
| qLRL_1_1 | LRL | S1H_498200346 | G/A | 1H | 498.20 | 0.08 | 3.23 |
|  |  | S1H_498516564 | C/A | 1H | 498.52 | 0.09 | 3.93 |
|  |  | S1H_498665554 | C/T | 1H | 498.67 | 0.09 | 3.87 |
|  |  | S1H_498665616 | T/C | 1H | 498.67 | 0.08 | 3.30 |
|  |  | S1H_498665635 | T/C | 1H | 498.67 | 0.08 | 3.30 |
|  |  | S1H_498665649 | G/A | 1H | 498.67 | 0.09 | 4.04 |
|  |  | S1H_500582726 | C/T | 1H | 500.58 | 0.07 | 2.83 |
|  |  | S1H_501363580 | T/C | 1H | 501.36 | 0.07 | 2.84 |
|  |  | S1H_503318377 | T/C | 1H | 503.32 | 0.07 | 2.93 |
|  |  | S1H_506584519 | T/A | 1H | 506.58 | 0.07 | 2.84 |
| qDCL_thick_2_1 | DCL_thick | S2H_710955052 | C/A | 2H | 710.96 | 0.30 | 2.93 |
|  |  | UNEAK-GBS-SNP-1492 | G/T | 2H | 710.96 | 0.30 | 2.74 |
|  |  | S2H_710993912 | T/C | 2H | 710.99 | 0.30 | 2.93 |
|  |  | S2H_711089145 | A/G | 2H | 711.09 | 0.30 | 2.82 |
|  |  | S2H_712332217 | A/G | 2H | 712.33 | 0.30 | 2.94 |
|  |  | S2H_713055971 | T/C | 2H | 713.06 | 0.30 | 3.06 |
|  |  | S2H_713055972 | C/T | 2H | 713.06 | 0.30 | 3.06 |
|  |  | S2H_713239978 | T/C | 2H | 713.24 | 0.30 | 3.05 |
|  |  | S2H_713239989 | A/G | 2H | 713.24 | 0.30 | 3.05 |
|  |  | S2H_714780815 | A/G | 2H | 714.78 | 0.29 | 3.09 |
|  |  | UNEAK-GBS-SNP-1501 | A/G | 2H | 714.78 | 0.29 | 2.86 |
|  |  | S2H_714859439 | T/C | 2H | 714.86 | 0.30 | 3.19 |
|  |  | S2H_716479144 | T/C | 2H | 716.48 | 0.31 | 3.48 |
|  |  | S2H_716541933 | T/C | 2H | 716.54 | 0.31 | 3.48 |
|  |  | UNEAK-GBS-SNP-1509 | T/C | 2H | 716.54 | 0.31 | 3.36 |
|  |  | S2H_716879136 | G/C | 2H | 716.88 | 0.29 | 2.73 |
|  |  | S2H_716879150 | A/G | 2H | 716.88 | 0.29 | 2.73 |
|  |  | UNEAK-GBS-SNP-1514 | C/T | 2H | 718.47 | 0.30 | 2.77 |
|  |  | S2H_718986613 | G/A | 2H | 718.99 | 0.30 | 3.20 |
|  |  | S2H_719046568 | G/A | 2H | 719.05 | 0.31 | 3.35 |
|  |  | UNEAK-GBS-SNP-1520 | T/G | 2H | 719.38 | 0.31 | 3.38 |
|  |  | S2H_719823613 | A/T | 2H | 719.82 | 0.30 | 2.82 |
|  |  | S2H_719823618 | T/C | 2H | 719.82 | 0.30 | 2.80 |
|  |  | S2H_719823646 | A/G | 2H | 719.82 | 0.30 | 2.82 |
| qRB_2_1 | RB | S2H_725794775 | A/G | 2H | 725.79 | 0.31 | 2.98 |
|  |  | S2H_725794873 | T/C | 2H | 725.79 | 0.31 | 2.78 |
|  |  | S2H_727648550 | C/T | 2H | 727.65 | 0.32 | 3.89 |
|  |  | S2H_727977246 | C/G | 2H | 727.98 | 0.31 | 2.73 |
|  |  | UNEAK-GBS-SNP-1546 | C/G | 2H | 727.98 | 0.31 | 3.38 |
|  |  | S2H_730572122 | A/G | 2H | 730.57 | 0.31 | 2.86 |
| qRD_20_2_1 | RD_20 | S2H_239094260 | A/G | 2H | 239.09 | 0.07 | 3.18 |
|  |  | UNEAK-GBS-SNP-1070 | A/G | 2H | 239.09 | 0.07 | 3.03 |
|  |  | UNEAK-GBS-SNP-1074 | C/A | 2H | 243.69 | 0.17 | 9.34 |
|  |  | S2H_246219296 | C/G | 2H | 246.22 | 0.07 | 3.17 |
|  |  | UNEAK-GBS-SNP-1076 | C/G | 2H | 246.22 | 0.07 | 3.19 |
| qTRL_2_1 | TRL | S2H_714780815 | A/G | 2H | 714.78 | 0.15 | 2.92 |
|  |  | S2H_714859439 | T/C | 2H | 714.86 | 0.17 | 2.82 |
|  |  | S2H_716479144 | T/C | 2H | 716.48 | 0.18 | 3.07 |
|  |  | S2H_716541933 | T/C | 2H | 716.54 | 0.18 | 3.07 |
|  |  | UNEAK-GBS-SNP-1509 | T/C | 2H | 716.54 | 0.17 | 2.92 |
|  |  | S2H_716879136 | G/C | 2H | 716.88 | 0.15 | 2.78 |
|  |  | S2H_716879150 | A/G | 2H | 716.88 | 0.15 | 2.78 |
|  |  | S2H_718986613 | G/A | 2H | 718.99 | 0.17 | 2.81 |
|  |  | S2H_719046568 | G/A | 2H | 719.05 | 0.18 | 3.01 |
|  |  | UNEAK-GBS-SNP-1520 | T/G | 2H | 719.38 | 0.17 | 2.92 |
|  |  | S2H_721941968 | G/C | 2H | 721.94 | 0.16 | 2.72 |
|  |  | S2H_721942017 | G/C | 2H | 721.94 | 0.16 | 2.72 |
| qRL_40_2_1 | RL_40 | S2H_714915633 | G/T | 2H | 714.92 | 0.09 | 2.83 |
|  |  | UNEAK-GBS-SNP-1503 | G/T | 2H | 714.92 | 0.10 | 3.21 |
|  |  | S2H_715705260 | A/G | 2H | 715.71 | 0.10 | 3.48 |
|  |  | S2H_716479144 | T/C | 2H | 716.48 | 0.10 | 3.10 |
|  |  | S2H_716541933 | T/C | 2H | 716.54 | 0.10 | 3.10 |
|  |  | UNEAK-GBS-SNP-1514 | C/T | 2H | 718.47 | 0.10 | 3.10 |
|  |  | S2H_718986613 | G/A | 2H | 718.99 | 0.10 | 3.26 |
|  |  | S2H_719046568 | G/A | 2H | 719.05 | 0.11 | 3.48 |
|  |  | UNEAK-GBS-SNP-1520 | T/G | 2H | 719.38 | 0.11 | 3.43 |
|  |  | S2H_721194317 | G/A | 2H | 721.19 | 0.10 | 3.27 |
|  |  | S2H_721194693 | C/G | 2H | 721.19 | 0.10 | 3.26 |
|  |  | S2H_721194706 | T/C | 2H | 721.19 | 0.11 | 3.49 |
|  |  | S2H_721941968 | G/C | 2H | 721.94 | 0.10 | 3.27 |
|  |  | S2H_721942017 | G/C | 2H | 721.94 | 0.10 | 3.27 |
|  |  | S2H_723096995 | C/T | 2H | 723.10 | 0.11 | 3.59 |
|  |  | S2H_723509063 | T/G | 2H | 723.51 | 0.11 | 3.61 |
|  |  | UNEAK-GBS-SNP-1526 | T/G | 2H | 723.51 | 0.11 | 3.85 |
|  |  | S2H_724944656 | T/C | 2H | 724.94 | 0.10 | 2.96 |
|  |  | S2H_724944659 | T/C | 2H | 724.94 | 0.10 | 2.96 |
|  |  | S2H_725104552 | C/T | 2H | 725.10 | 0.10 | 3.56 |
| qSRL_2_1 | SRL | S2H_716479144 | T/C | 2H | 716.48 | 0.17 | 2.91 |
|  |  | S2H_716541933 | T/C | 2H | 716.54 | 0.17 | 2.91 |
|  |  | UNEAK-GBS-SNP-1509 | T/C | 2H | 716.54 | 0.17 | 2.74 |
|  |  | S2H_716879136 | G/C | 2H | 716.88 | 0.17 | 3.19 |
|  |  | S2H_716879150 | A/G | 2H | 716.88 | 0.17 | 3.19 |
|  |  | S2H_717280839 | G/A | 2H | 717.28 | 0.16 | 2.73 |
|  |  | S2H_717280859 | G/C | 2H | 717.28 | 0.16 | 2.73 |
|  |  | S2H_719046568 | G/A | 2H | 719.05 | 0.17 | 2.90 |
|  |  | UNEAK-GBS-SNP-1520 | T/G | 2H | 719.38 | 0.17 | 2.79 |
| qSH_2_1 | SH | S2H_42238865 | A/G | 2H | 42.24 | 0.32 | 2.90 |
|  |  | S2H_42675430 | C/T | 2H | 42.68 | 0.33 | 3.29 |
|  |  | S2H_42675447 | C/A | 2H | 42.68 | 0.33 | 3.29 |
|  |  | S2H_43862226 | C/T | 2H | 43.86 | 0.33 | 3.28 |
|  |  | UNEAK-GBS-SNP-855 | G/A | 2H | 43.86 | 0.33 | 3.46 |
|  |  | S2H_44537519 | G/T | 2H | 44.54 | 0.34 | 3.91 |
|  |  | UNEAK-GBS-SNP-856 | C/A | 2H | 44.54 | 0.34 | 3.76 |
|  |  | S2H_44541927 | G/A | 2H | 44.54 | 0.34 | 4.23 |
|  |  | S2H_44543906 | A/C | 2H | 44.54 | 0.34 | 3.70 |
|  |  | S2H_45108524 | A/G | 2H | 45.11 | 0.32 | 2.99 |
| qRB_40_3_1 | RB_40 | S3H_609690640 | G/A | 3H | 609.69 | 0.16 | 2.82 |
|  |  | S3H_609690658 | A/G | 3H | 609.69 | 0.16 | 2.82 |
|  |  | S3H_614819853 | T/C | 3H | 614.82 | 0.15 | 3.28 |
|  |  | S3H_614819873 | C/T | 3H | 614.82 | 0.15 | 3.28 |
|  |  | S3H_614819882 | C/G | 3H | 614.82 | 0.15 | 3.28 |
| qRD_20_3_1 | RD_20 | S3H_629447254 | C/T | 3H | 629.45 | 0.10 | 3.19 |
|  |  | S3H_629447277 | C/T | 3H | 629.45 | 0.10 | 3.19 |
|  |  | UNEAK-GBS-SNP-2243 | C/A | 3H | 631.07 | 0.10 | 3.19 |
|  |  | UNEAK-GBS-SNP-2244 | T/A | 3H | 631.22 | 0.10 | 3.19 |
|  |  | S3H_634086093 | C/T | 3H | 634.09 | 0.20 | 14.24 |
| qRD_20_3_2 |  | S3H_681143774 | G/T | 3H | 681.14 | 0.10 | 3.19 |
|  |  | S3H_681484846 | C/T | 3H | 681.48 | 0.10 | 3.19 |
|  |  | S3H_686750146 | G/A | 3H | 686.75 | 0.10 | 3.11 |
|  |  | S3H_686750162 | C/T | 3H | 686.75 | 0.10 | 3.11 |
|  |  | S3H_687148930 | C/T | 3H | 687.15 | 0.09 | 2.86 |
| qRD_top_3_1 | RD_top | S3H_628916492 | T/G | 3H | 628.92 | 0.12 | 2.86 |
|  |  | S3H_629146627 | C/G | 3H | 629.15 | 0.12 | 2.73 |
|  |  | S3H_629447254 | C/T | 3H | 629.45 | 0.18 | 4.12 |
|  |  | S3H_629447277 | C/T | 3H | 629.45 | 0.18 | 4.12 |
|  |  | S3H_629938591 | T/G | 3H | 629.94 | 0.14 | 2.76 |
|  |  | UNEAK-GBS-SNP-2243 | C/A | 3H | 631.07 | 0.18 | 4.12 |
|  |  | UNEAK-GBS-SNP-2244 | T/A | 3H | 631.22 | 0.18 | 4.12 |
|  |  | UNEAK-GBS-SNP-2246 | C/G | 3H | 631.89 | 0.16 | 4.08 |
|  |  | S3H_634086093 | C/T | 3H | 634.09 | 0.29 | 17.88 |
| qRD_top_3_2 |  | S3H_681143774 | G/T | 3H | 681.14 | 0.18 | 4.12 |
|  |  | S3H_681484846 | C/T | 3H | 681.48 | 0.18 | 4.12 |
|  |  | S3H_682742320 | T/G | 3H | 682.74 | 0.12 | 3.30 |
|  |  | S3H_683347874 | G/A | 3H | 683.35 | 0.12 | 3.14 |
|  |  | S3H_688604698 | T/C | 3H | 688.60 | 0.24 | 6.55 |
| qRD_top_3_3 |  | S3H_693813220 | C/T | 3H | 693.81 | 0.18 | 4.12 |
|  |  | S3H_694179554 | C/T | 3H | 694.18 | 0.14 | 2.88 |
|  |  | UNEAK-GBS-SNP-2417 | C/T | 3H | 694.18 | 0.14 | 3.11 |
|  |  | S3H_694202124 | C/T | 3H | 694.20 | 0.14 | 3.16 |
|  |  | S3H_694362876 | G/C | 3H | 694.36 | 0.14 | 2.87 |
|  |  | UNEAK-GBS-SNP-2418 | C/G | 3H | 694.36 | 0.14 | 2.80 |
| qLRL_4_1 | LRL | S4H_621551402 | G/A | 4H | 621.55 | 0.10 | 3.34 |
|  |  | UNEAK-GBS-SNP-3115 | G/A | 4H | 621.55 | 0.09 | 3.02 |
|  |  | S4H_623322042 | A/C | 4H | 623.32 | 0.09 | 3.70 |
|  |  | UNEAK-GBS-SNP-3121 | A/C | 4H | 623.32 | 0.09 | 3.70 |
|  |  | UNEAK-GBS-SNP-3122 | A/C | 4H | 623.32 | 0.09 | 3.50 |
|  |  | S4H_623448342 | A/C | 4H | 623.45 | 0.10 | 2.86 |
|  |  | S4H_623514633 | T/C | 4H | 623.51 | 0.09 | 2.88 |
|  |  | S4H_624588478 | G/A | 4H | 624.59 | 0.07 | 3.05 |
|  |  | UNEAK-GBS-SNP-3126 | T/C | 4H | 624.59 | 0.08 | 3.13 |
|  |  | UNEAK-GBS-SNP-3127 | T/C | 4H | 624.59 | 0.09 | 3.52 |
|  |  | S4H_624589381 | T/C | 4H | 624.59 | 0.09 | 3.30 |
|  |  | S4H_624627799 | G/T | 4H | 624.63 | 0.09 | 3.32 |
|  |  | S4H_625170580 | A/G | 4H | 625.17 | 0.09 | 3.30 |
|  |  | S4H_625311528 | T/A | 4H | 625.31 | 0.08 | 2.74 |
|  |  | UNEAK-GBS-SNP-3131 | A/T | 4H | 625.31 | 0.07 | 2.73 |
|  |  | S4H_626405290 | G/C | 4H | 626.41 | 0.08 | 2.74 |
|  |  | S4H_626731016 | G/A | 4H | 626.73 | 0.08 | 2.77 |
|  |  | S4H_626731088 | T/A | 4H | 626.73 | 0.08 | 2.74 |
| qRD_top_4_1 | RD_top | S4H_9112201 | G/A | 4H | 9.11 | 0.13 | 3.20 |
|  |  | UNEAK-GBS-SNP-2458 | A/C | 4H | 9.11 | 0.12 | 2.90 |
|  |  | S4H_9577892 | G/A | 4H | 9.58 | 0.12 | 3.02 |
|  |  | S4H_9577893 | T/G | 4H | 9.58 | 0.12 | 3.02 |
|  |  | S4H_9779812 | G/C | 4H | 9.78 | 0.18 | 3.97 |
| qRL_lower_4_1 | RL_lower | S4H_531521393 | A/G | 4H | 531.52 | 0.36 | 2.89 |
|  |  | S4H_531547174 | C/T | 4H | 531.55 | 0.36 | 2.80 |
|  |  | S4H_531547205 | G/T | 4H | 531.55 | 0.36 | 2.80 |
|  |  | S4H_533098639 | G/A | 4H | 533.10 | 0.36 | 2.84 |
|  |  | S4H_534648918 | G/A | 4H | 534.65 | 0.36 | 2.73 |
| qTill_4_1 | Till | S4H_69301219 | G/T | 4H | 69.30 | 0.16 | 2.74 |
|  |  | S4H_73227377 | A/G | 4H | 73.23 | 0.16 | 2.74 |
|  |  | S4H_73227388 | T/G | 4H | 73.23 | 0.16 | 2.74 |
|  |  | S4H_73227414 | T/C | 4H | 73.23 | 0.16 | 2.74 |
|  |  | S4H_79132829 | T/C | 4H | 79.13 | 0.16 | 2.74 |
| qDCL_thin_5_1 | DCL_thin | S5H_586681271 | T/G | 5H | 586.68 | 0.07 | 3.48 |
|  |  | S5H_587707182 | G/A | 5H | 587.71 | 0.09 | 3.98 |
|  |  | S5H_596702405 | C/G | 5H | 596.70 | 0.09 | 3.55 |
|  |  | S5H_597066960 | C/T | 5H | 597.07 | 0.08 | 3.43 |
|  |  | S5H_597066987 | A/T | 5H | 597.07 | 0.09 | 3.90 |
|  |  | S5H_597069839 | A/G | 5H | 597.07 | 0.09 | 3.81 |
|  |  | S5H_597069857 | T/C | 5H | 597.07 | 0.09 | 3.81 |
|  |  | UNEAK-GBS-SNP-3926 | A/G | 5H | 597.07 | 0.08 | 3.59 |
| qDCL_thin_5_2 |  | S5H_645157956 | G/T | 5H | 645.16 | 0.10 | 3.44 |
|  |  | S5H_645157959 | C/T | 5H | 645.16 | 0.10 | 3.44 |
|  |  | S5H_645158922 | T/C | 5H | 645.16 | 0.11 | 3.99 |
|  |  | S5H_645158945 | T/C | 5H | 645.16 | 0.08 | 3.45 |
|  |  | S5H_645158965 | A/T | 5H | 645.16 | 0.10 | 3.66 |
|  |  | S5H_645158966 | T/C | 5H | 645.16 | 0.10 | 3.66 |
|  |  | S5H_646375764 | G/C | 5H | 646.38 | 0.08 | 3.65 |
|  |  | S5H_646375766 | A/G | 5H | 646.38 | 0.08 | 3.65 |
|  |  | S5H_646968261 | T/A | 5H | 646.97 | 0.09 | 3.04 |
|  |  | S5H_646968262 | A/G | 5H | 646.97 | 0.08 | 3.20 |
| qLRL_5_1 | LRL | S5H_596702405 | C/G | 5H | 596.70 | 0.10 | 3.29 |
|  |  | S5H_597066960 | C/T | 5H | 597.07 | 0.08 | 2.98 |
|  |  | S5H_597066987 | A/T | 5H | 597.07 | 0.10 | 3.64 |
|  |  | S5H_597069839 | A/G | 5H | 597.07 | 0.10 | 3.60 |
|  |  | S5H_597069857 | T/C | 5H | 597.07 | 0.10 | 3.60 |
|  |  | UNEAK-GBS-SNP-3926 | A/G | 5H | 597.07 | 0.09 | 3.22 |
|  |  | UNEAK-GBS-SNP-3927 | G/A | 5H | 597.36 | 0.09 | 3.40 |
| qLRL_5_2 |  | S5H_645157956 | G/T | 5H | 645.16 | 0.09 | 2.78 |
|  |  | S5H_645157959 | C/T | 5H | 645.16 | 0.09 | 2.78 |
|  |  | S5H_645158922 | T/C | 5H | 645.16 | 0.12 | 3.84 |
|  |  | S5H_645158945 | T/C | 5H | 645.16 | 0.08 | 2.99 |
|  |  | S5H_645158965 | A/T | 5H | 645.16 | 0.10 | 2.97 |
|  |  | S5H_645158966 | T/C | 5H | 645.16 | 0.10 | 2.97 |
|  |  | S5H_646375764 | G/C | 5H | 646.38 | 0.07 | 2.97 |
|  |  | S5H_646375766 | A/G | 5H | 646.38 | 0.07 | 2.97 |
|  |  | S5H_646968261 | T/A | 5H | 646.97 | 0.10 | 2.98 |
|  |  | S5H_646968262 | A/G | 5H | 646.97 | 0.08 | 2.83 |
| qRB_5_1 | RB | S5H_626687939 | C/G | 5H | 626.69 | 0.30 | 2.74 |
|  |  | UNEAK-GBS-SNP-4002 | G/C | 5H | 626.69 | 0.30 | 2.72 |
|  |  | S5H_629052593 | C/T | 5H | 629.05 | 0.32 | 3.72 |
|  |  | S5H_629052627 | C/G | 5H | 629.05 | 0.32 | 3.72 |
|  |  | S5H_629189593 | G/A | 5H | 629.19 | 0.30 | 2.77 |
|  |  | S5H_634513773 | G/A | 5H | 634.51 | 0.31 | 3.20 |
|  |  | S5H_636675975 | C/T | 5H | 636.68 | 0.31 | 3.05 |
| qRLR_5_1 | RLR | S5H_556785160 | C/T | 5H | 556.79 | 0.10 | 2.77 |
|  |  | S5H_557743377 | T/C | 5H | 557.74 | 0.13 | 3.18 |
|  |  | S5H_557750447 | G/C | 5H | 557.75 | 0.12 | 2.76 |
|  |  | S5H_557860404 | C/T | 5H | 557.86 | 0.12 | 2.97 |
|  |  | S5H_558300461 | T/C | 5H | 558.30 | 0.12 | 3.07 |
|  |  | S5H_559347678 | G/T | 5H | 559.35 | 0.11 | 2.89 |
|  |  | S5H_562933563 | C/G | 5H | 562.93 | 0.11 | 3.85 |
|  |  | UNEAK-GBS-SNP-3833 | G/C | 5H | 562.93 | 0.11 | 3.82 |
| qDCL_thick_7_1 | DCL_thick | S7H_640721887 | C/T | 7H | 614.23 | 0.31 | 3.46 |
|  |  | UNEAK-GBS-SNP-5917 | C/A | 7H | 614.23 | 0.31 | 3.46 |
|  |  | S7H_646893702 | C/A | 7H | 615.10 | 0.31 | 3.46 |
|  |  | S7H_648943940 | T/C | 7H | 615.10 | 0.31 | 3.46 |
|  |  | S7H_648943985 | G/A | 7H | 621.46 | 0.30 | 2.75 |
| qDCL_thick_7_2 |  | S7H_649584613 | A/T | 7H | 640.72 | 0.30 | 2.74 |
|  |  | S7H_640721887 | C/T | 7H | 640.72 | 0.30 | 2.74 |
|  |  | UNEAK-GBS-SNP-5917 | C/A | 7H | 646.89 | 0.30 | 2.90 |
|  |  | S7H_646893702 | C/A | 7H | 646.89 | 0.30 | 2.90 |
|  |  | S7H_648943940 | T/C | 7H | 648.94 | 0.30 | 2.90 |
|  |  | S7H_648943985 | G/A | 7H | 648.94 | 0.30 | 2.90 |
|  |  | S7H_649584590 | T/C | 7H | 649.58 | 0.30 | 2.90 |
|  |  | S7H_649584613 | A/T | 7H | 649.58 | 0.30 | 2.90 |
| qLRL_7_1 | LRL | S7H_600029406 | G/T | 7H | 600.03 | 0.07 | 3.05 |
|  |  | UNEAK-GBS-SNP-5783 | C/A | 7H | 600.03 | 0.08 | 3.15 |
|  |  | S7H_600148873 | G/T | 7H | 600.15 | 0.08 | 3.39 |
|  |  | S7H_604224559 | G/T | 7H | 604.22 | 0.09 | 2.78 |
|  |  | S7H_604224594 | G/A | 7H | 604.22 | 0.09 | 2.78 |
| qRA_7_1 | RA | S7H_614231925 | T/C | 7H | 614.23 | 0.19 | 3.42 |
|  |  | S7H_614231977 | G/A | 7H | 614.23 | 0.19 | 3.42 |
|  |  | S7H_615099417 | A/C | 7H | 615.10 | 0.19 | 3.42 |
|  |  | S7H_615099449 | T/C | 7H | 615.10 | 0.19 | 3.42 |
|  |  | S7H_624659469 | C/T | 7H | 624.66 | 0.20 | 2.95 |
| qRB_7_1 | RB | S7H_34359178 | C/T | 7H | 34.36 | 0.32 | 3.07 |
|  |  | S7H_34398528 | C/T | 7H | 34.40 | 0.32 | 3.13 |
|  |  | S7H_34821156 | G/C | 7H | 34.82 | 0.32 | 3.07 |
|  |  | S7H_34821165 | A/C | 7H | 34.82 | 0.32 | 3.07 |
|  |  | S7H_34914954 | G/A | 7H | 34.91 | 0.32 | 3.07 |
|  |  | S7H_34914984 | T/C | 7H | 34.91 | 0.32 | 3.07 |
| qR/S_7_1 | R/S | S7H_33038330 | A/G | 7H | 33.04 | 0.29 | 2.85 |
|  |  | UNEAK-GBS-SNP-5265 | T/C | 7H | 33.04 | 0.29 | 2.71 |
|  |  | S7H_34359178 | C/T | 7H | 34.36 | 0.30 | 2.75 |
|  |  | S7H_34398528 | C/T | 7H | 34.40 | 0.31 | 3.02 |
|  |  | S7H_34821156 | G/C | 7H | 34.82 | 0.30 | 2.75 |
|  |  | S7H_34821165 | A/C | 7H | 34.82 | 0.30 | 2.75 |
|  |  | S7H_34914954 | G/A | 7H | 34.91 | 0.30 | 2.75 |
|  |  | S7H_34914984 | T/C | 7H | 34.91 | 0.30 | 2.75 |
|  |  | S7H_34924908 | T/A | 7H | 34.92 | 0.29 | 3.13 |
|  |  | UNEAK-GBS-SNP-5271 | T/A | 7H | 34.92 | 0.30 | 3.06 |
|  |  | S7H_36124776 | T/C | 7H | 36.12 | 0.30 | 3.18 |
|  |  | S7H_36124815 | G/T | 7H | 36.12 | 0.30 | 3.18 |
|  |  | S7H_36921505 | G/A | 7H | 36.92 | 0.29 | 2.93 |
|  |  | S7H_37357544 | G/T | 7H | 37.36 | 0.30 | 3.55 |
|  |  | UNEAK-GBS-SNP-5274 | G/T | 7H | 37.36 | 0.30 | 3.80 |
| qRD_7_1 | RD | S7H_625140060 | G/A | 7H | 625.14 | 0.16 | 3.29 |
|  |  | S7H_625140066 | T/G | 7H | 625.14 | 0.16 | 3.29 |
|  |  | S7H_626403602 | G/T | 7H | 626.40 | 0.17 | 3.50 |
|  |  | S7H_626423098 | G/A | 7H | 626.42 | 0.17 | 3.78 |
|  |  | UNEAK-GBS-SNP-5846 | C/T | 7H | 626.42 | 0.16 | 3.24 |
|  |  | S7H_626423213 | A/T | 7H | 626.42 | 0.17 | 4.02 |
| qRD_top_7_1 | RD_top | S7H_37357536 | G/C | 7H | 37.36 | 0.14 | 3.03 |
|  |  | S7H_37729067 | T/C | 7H | 37.73 | 0.14 | 2.93 |
|  |  | UNEAK-GBS-SNP-5281 | T/G | 7H | 38.83 | 0.14 | 3.20 |
|  |  | S7H_39119968 | A/C | 7H | 39.12 | 0.13 | 2.99 |
|  |  | S7H_39119976 | G/C | 7H | 39.12 | 0.13 | 2.99 |
|  |  | UNEAK-GBS-SNP-5284 | A/C | 7H | 39.12 | 0.13 | 2.99 |
|  |  | S7H_39315484 | C/A | 7H | 39.32 | 0.14 | 3.44 |
|  |  | S7H_39315531 | T/C | 7H | 39.32 | 0.14 | 3.44 |
|  |  | S7H_39315536 | G/A | 7H | 39.32 | 0.14 | 3.44 |
|  |  | UNEAK-GBS-SNP-5288 | G/A | 7H | 40.45 | 0.14 | 3.27 |
|  |  | S7H_41884636 | A/G | 7H | 41.88 | 0.18 | 4.12 |
| qTRL_7_1 | TRL | UNEAK-GBS-SNP-5917 | C/A | 7H | 646.89 | 0.16 | 2.88 |
|  |  | S7H_646893702 | C/A | 7H | 646.89 | 0.16 | 2.88 |
|  |  | S7H_648033725 | C/A | 7H | 648.03 | 0.16 | 3.05 |
|  |  | S7H_648204335 | T/C | 7H | 648.20 | 0.16 | 3.05 |
|  |  | S7H_648204355 | G/C | 7H | 648.20 | 0.16 | 3.05 |
|  |  | UNEAK-GBS-SNP-5923 | C/G | 7H | 648.20 | 0.16 | 3.05 |
|  |  | S7H_648211886 | G/A | 7H | 648.21 | 0.16 | 3.05 |
|  |  | S7H_648211895 | T/C | 7H | 648.21 | 0.16 | 3.05 |
|  |  | S7H_648211922 | G/A | 7H | 648.21 | 0.16 | 3.05 |
|  |  | S7H_648402306 | G/C | 7H | 648.40 | 0.16 | 3.05 |
|  |  | S7H_648943940 | T/C | 7H | 648.94 | 0.16 | 2.88 |
|  |  | S7H_648943985 | G/A | 7H | 648.94 | 0.16 | 2.88 |
|  |  | S7H_649584590 | T/C | 7H | 649.58 | 0.16 | 2.88 |
|  |  | S7H_649584613 | A/T | 7H | 649.58 | 0.16 | 2.88 |
| qRL_40_7_1 | RL_40 | S7H_640721887 | C/T | 7H | 640.72 | 0.09 | 2.85 |
|  |  | UNEAK-GBS-SNP-5898 | G/A | 7H | 640.72 | 0.09 | 2.81 |
|  |  | S7H_648033725 | C/A | 7H | 648.03 | 0.09 | 2.71 |
|  |  | S7H_648204335 | T/C | 7H | 648.20 | 0.09 | 2.71 |
|  |  | S7H_648204355 | G/C | 7H | 648.20 | 0.09 | 2.71 |
|  |  | UNEAK-GBS-SNP-5923 | C/G | 7H | 648.20 | 0.09 | 2.71 |
|  |  | S7H_648211886 | G/A | 7H | 648.21 | 0.09 | 2.71 |
|  |  | S7H_648211895 | T/C | 7H | 648.21 | 0.09 | 2.71 |
|  |  | S7H_648211922 | G/A | 7H | 648.21 | 0.09 | 2.71 |
|  |  | S7H_648402306 | G/C | 7H | 648.40 | 0.09 | 2.71 |
| qRL_top_7_1 | RL_top | UNEAK-GBS-SNP-5914 | C/T | 7H | 645.73 | 0.23 | 3.76 |
|  |  | S7H_648033760 | C/A | 7H | 648.03 | 0.22 | 3.92 |
|  |  | S7H_648036258 | G/A | 7H | 648.04 | 0.22 | 3.97 |
|  |  | UNEAK-GBS-SNP-5921 | G/A | 7H | 648.04 | 0.22 | 3.80 |
|  |  | S7H_648402305 | G/C | 7H | 648.40 | 0.22 | 3.77 |
| qRLR_7_1 | RLR | S7H_612599532 | A/T | 7H | 612.60 | 0.09 | 2.89 |
|  |  | UNEAK-GBS-SNP-5808 | T/A | 7H | 612.78 | 0.10 | 3.31 |
|  |  | UNEAK-GBS-SNP-5809 | G/C | 7H | 612.78 | 0.10 | 2.99 |
|  |  | S7H_619472060 | T/C | 7H | 619.47 | 0.13 | 3.34 |
|  |  | S7H_619472073 | A/G | 7H | 619.47 | 0.13 | 3.34 |
| qRLR_7_2 |  | S7H_644461831 | T/C | 7H | 644.46 | 0.10 | 3.03 |
|  |  | S7H_645733480 | G/A | 7H | 645.73 | 0.18 | 3.67 |
|  |  | S7H_647501142 | T/C | 7H | 647.50 | 0.10 | 3.47 |
|  |  | UNEAK-GBS-SNP-5919 | T/C | 7H | 647.50 | 0.10 | 3.12 |
|  |  | S7H_648033762 | C/A | 7H | 648.03 | 0.10 | 2.92 |
|  |  | S7H_649651443 | C/G | 7H | 649.65 | 0.10 | 2.87 |
|  |  | UNEAK-GBS-SNP-5929 | G/A | 7H | 649.72 | 0.12 | 3.30 |
|  |  | S7H_651100183 | G/C | 7H | 651.10 | 0.18 | 3.67 |
|  |  | S7H_651177584 | T/C | 7H | 651.18 | 0.18 | 3.67 |
|  |  | S7H_651219003 | A/G | 7H | 651.22 | 0.18 | 3.67 |
|  |  | S7H_651483435 | G/C | 7H | 651.48 | 0.18 | 3.67 |
|  |  | S7H_653233132 | T/C | 7H | 653.23 | 0.16 | 2.78 |
| qRV_7_1 | RV | S7H_614231925 | T/C | 7H | 614.23 | 0.19 | 3.02 |
|  |  | S7H_614231977 | G/A | 7H | 614.23 | 0.19 | 3.02 |
|  |  | S7H_615099417 | A/C | 7H | 615.10 | 0.19 | 3.02 |
|  |  | S7H_615099449 | T/C | 7H | 615.10 | 0.19 | 3.02 |
|  |  | S7H_624659469 | C/T | 7H | 624.66 | 0.20 | 2.80 |
| qSH_7_1 | SH | S7H_624556104 | C/A | 7H | 624.56 | 0.31 | 2.97 |
|  |  | S7H_629580988 | C/T | 7H | 629.58 | 0.32 | 2.93 |
|  |  | S7H_629580989 | A/G | 7H | 629.58 | 0.32 | 2.93 |
|  |  | S7H_629581027 | G/T | 7H | 629.58 | 0.32 | 2.93 |
|  |  | S7H_629582513 | G/A | 7H | 629.58 | 0.32 | 3.33 |
|  |  | S7H_629582539 | G/A | 7H | 629.58 | 0.32 | 3.33 |
|  |  | S7H_629582551 | G/A | 7H | 629.58 | 0.32 | 3.33 |

* The trait codes are specified in Table 1.

** The significant GBS-SNPs associated with the traits were categorized based on their location in a similar physical region (Mb) in barley genome.

*** The physical location is the start of an interval of the genomic locations of strongly associated GBS-SNPs.
