# Supplementary figures and images for: Genetic characterization of root architectural traits in barley (Hordeum vulgare L.) using SNP markers
Source: Front Plant Sci. 2023 Oct 4;14:1265925. doi: 10.3389/fpls.2023.1265925 (PMC10582755; doi:10.3389/fpls.2023.1265925)

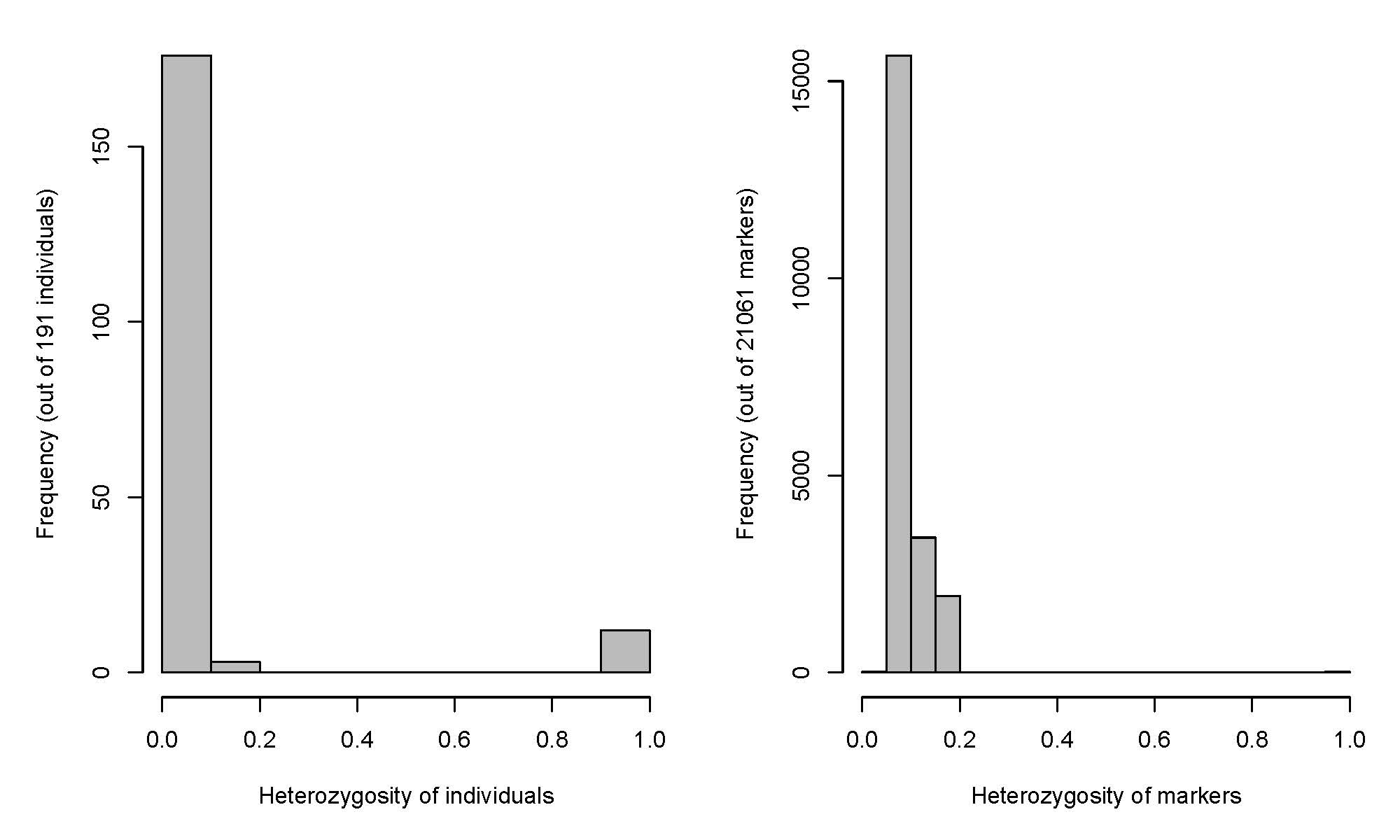

Supplement: Supplementary Figure 1 — Heterozygosity among 191 genotypes and GBS-SNPs used in the current study. The values on the X-axis indicate heterozygous allele proportions ranging from 0 to 1 in the association mapping panel. [file DataSheet_1.zip › Suppl Figures/Suppl Figure 1.docx]

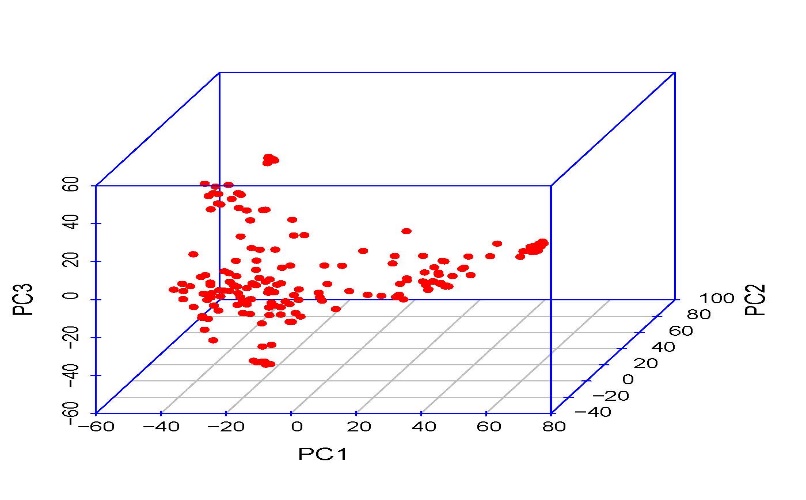

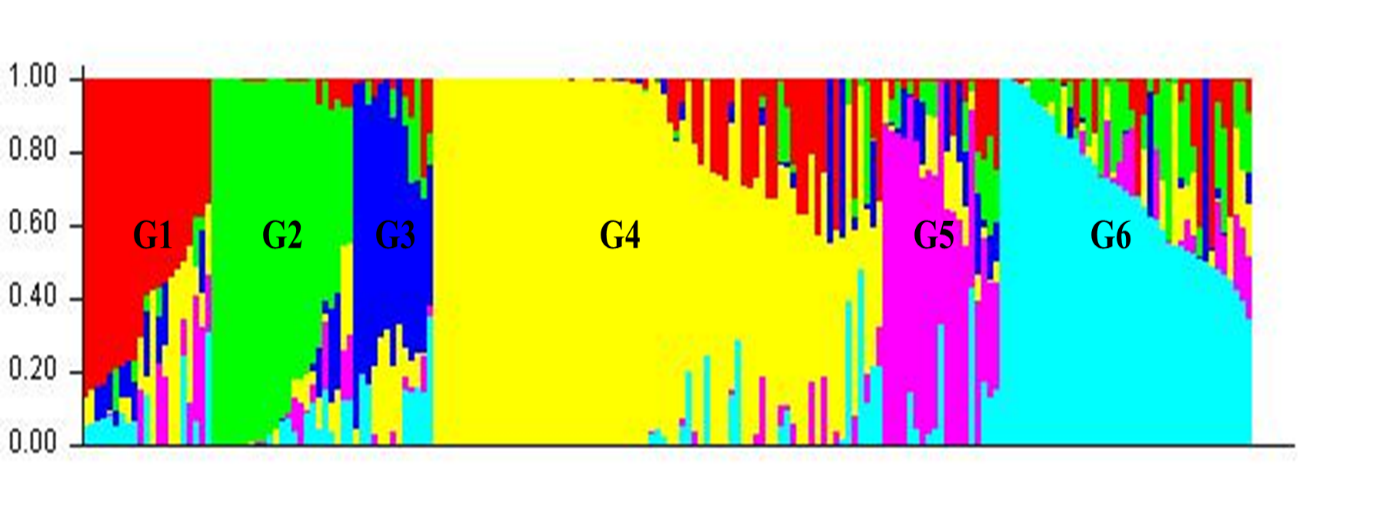

Supplement: Supplementary Figure 1 — Heterozygosity among 191 genotypes and GBS-SNPs used in the current study. The values on the X-axis indicate heterozygous allele proportions ranging from 0 to 1 in the association mapping panel. [file DataSheet_1.zip › Suppl Figures/Suppl Figure 2.docx]

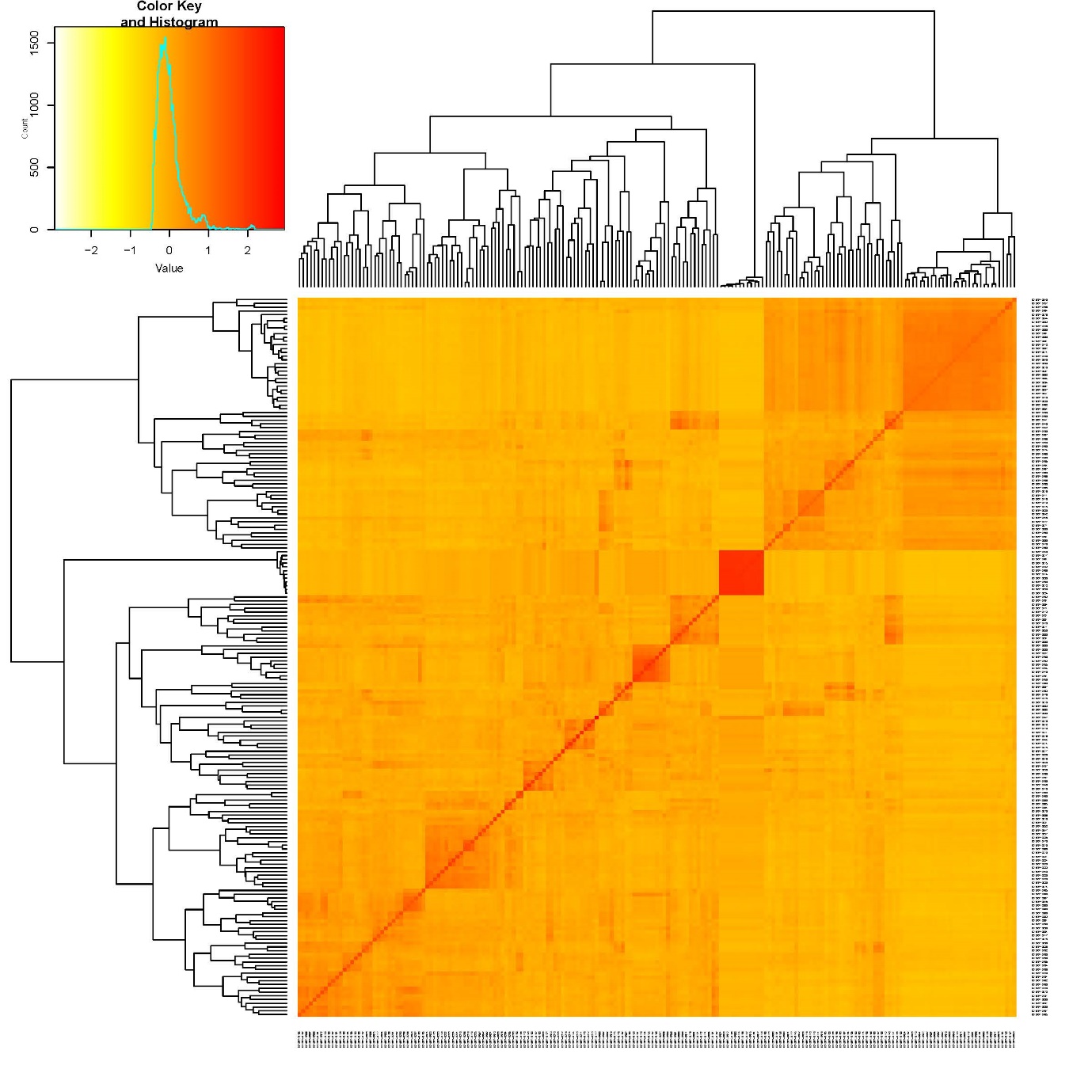

Supplement: Supplementary Figure 1 — Heterozygosity among 191 genotypes and GBS-SNPs used in the current study. The values on the X-axis indicate heterozygous allele proportions ranging from 0 to 1 in the association mapping panel. [file DataSheet_1.zip › Suppl Figures/Suppl Figure 3.docx]

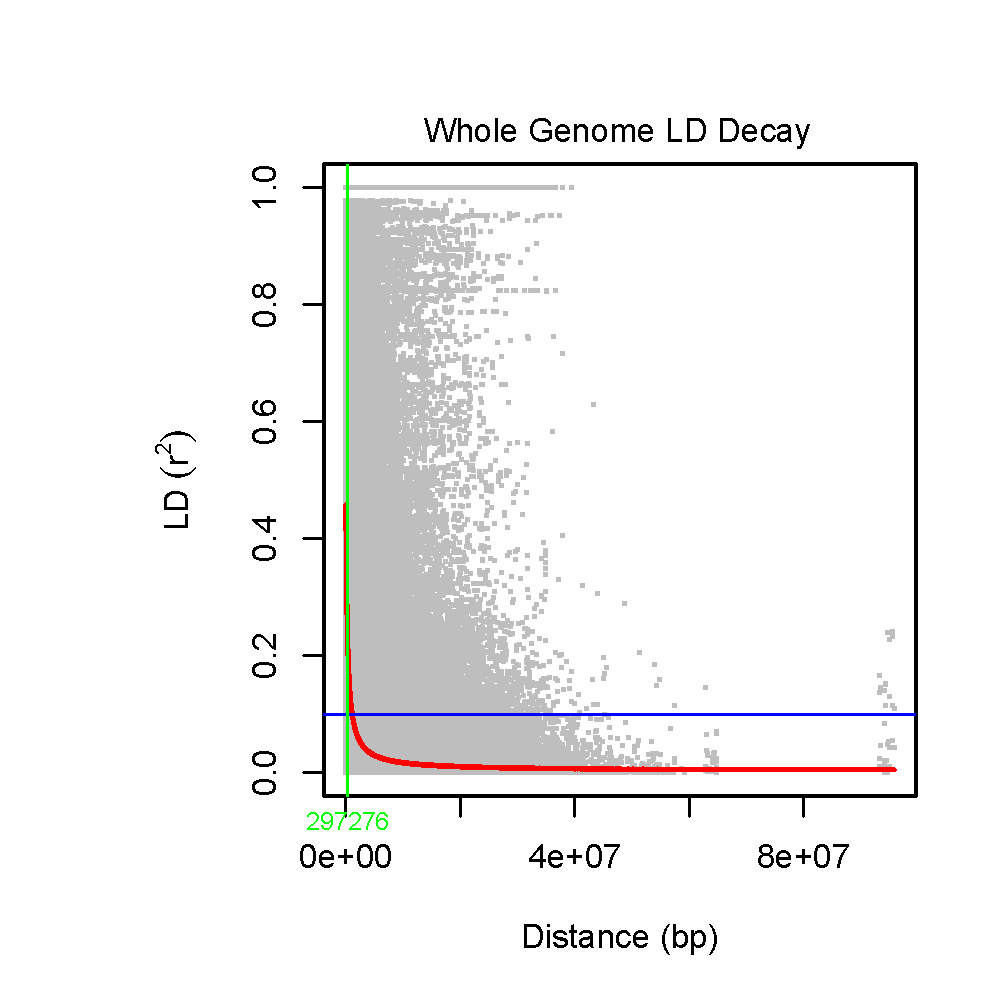


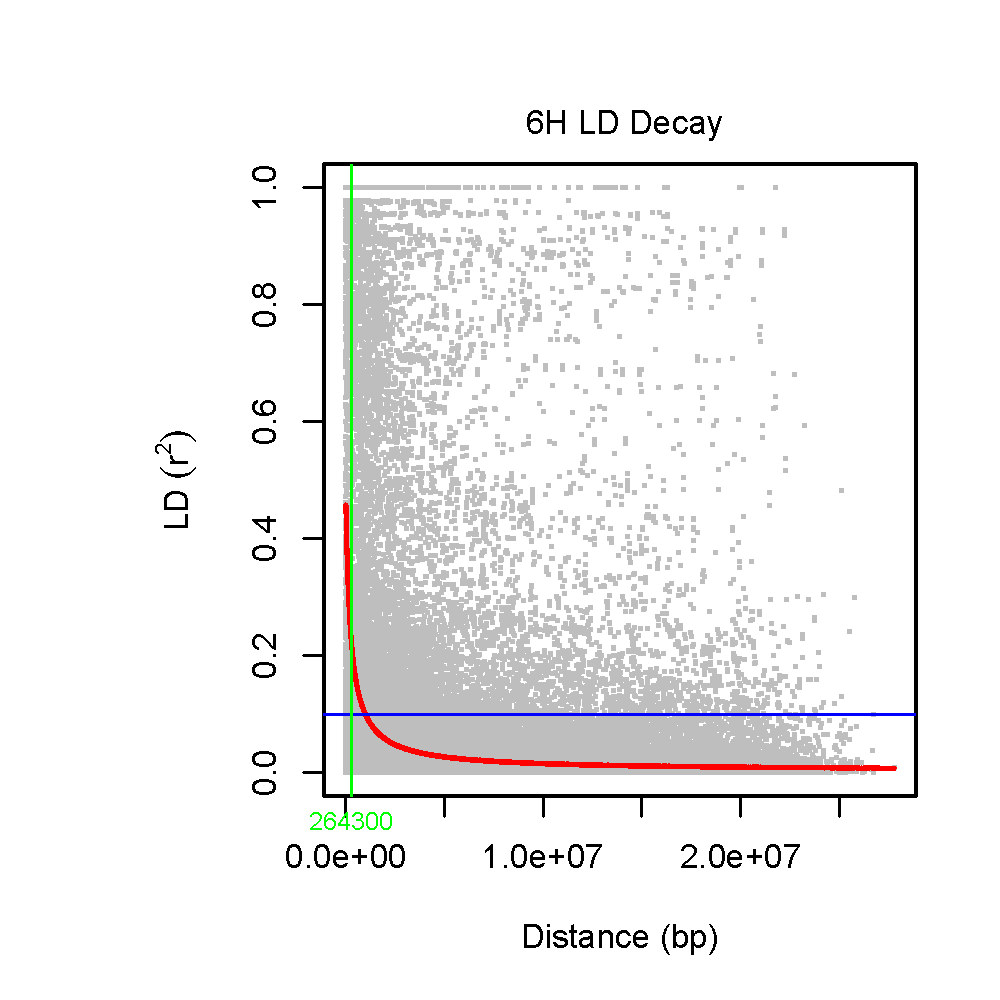

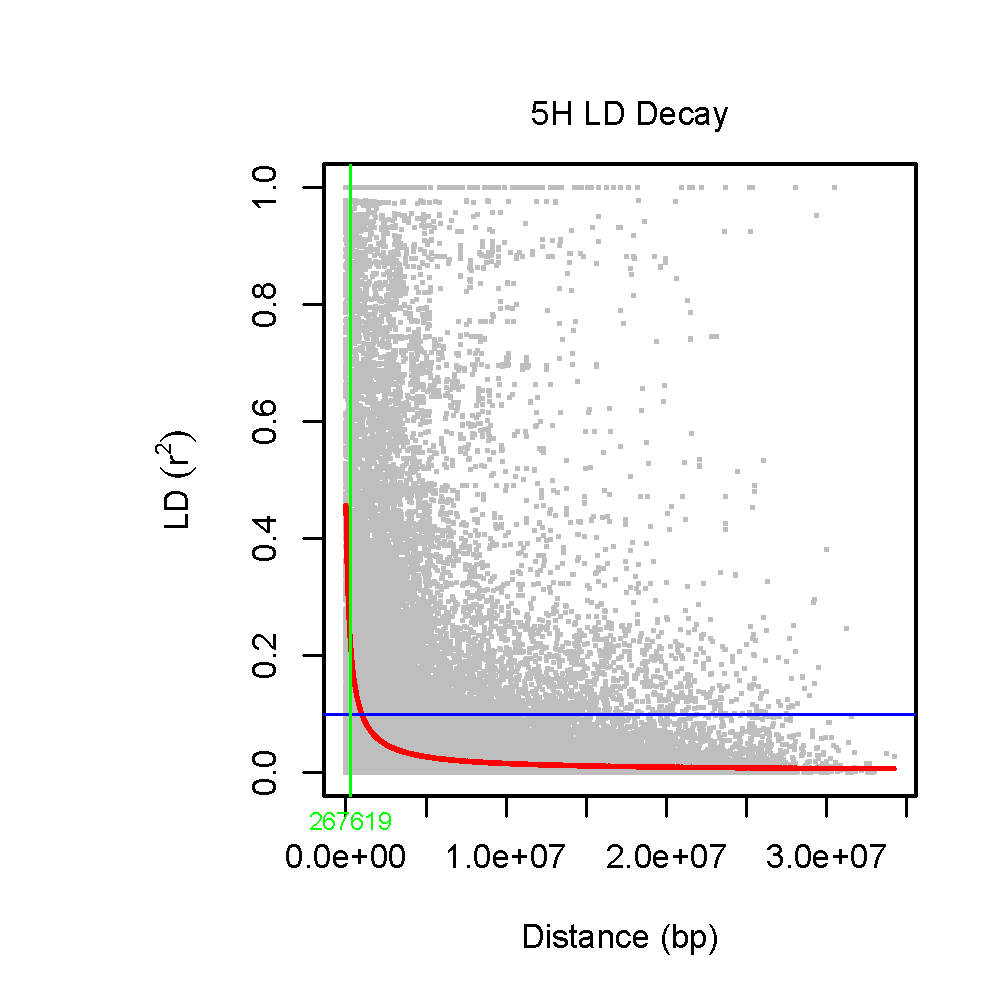

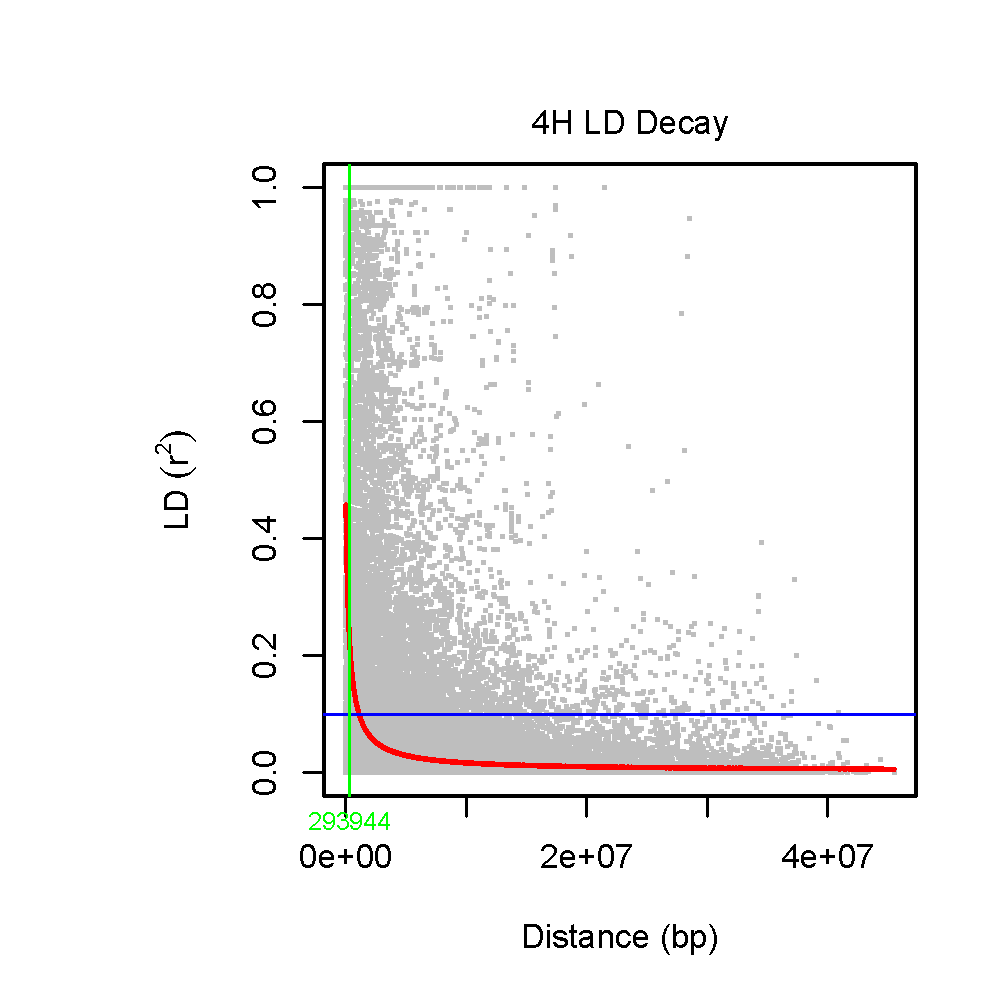

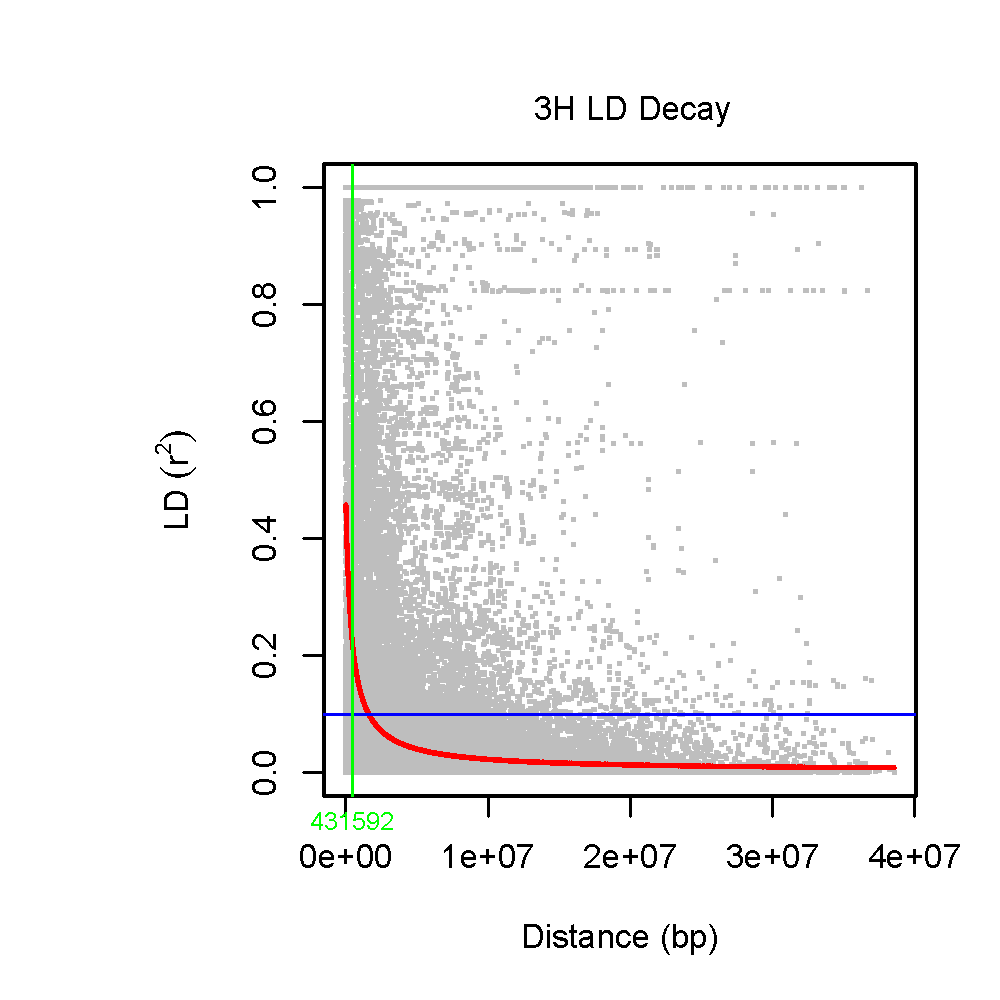

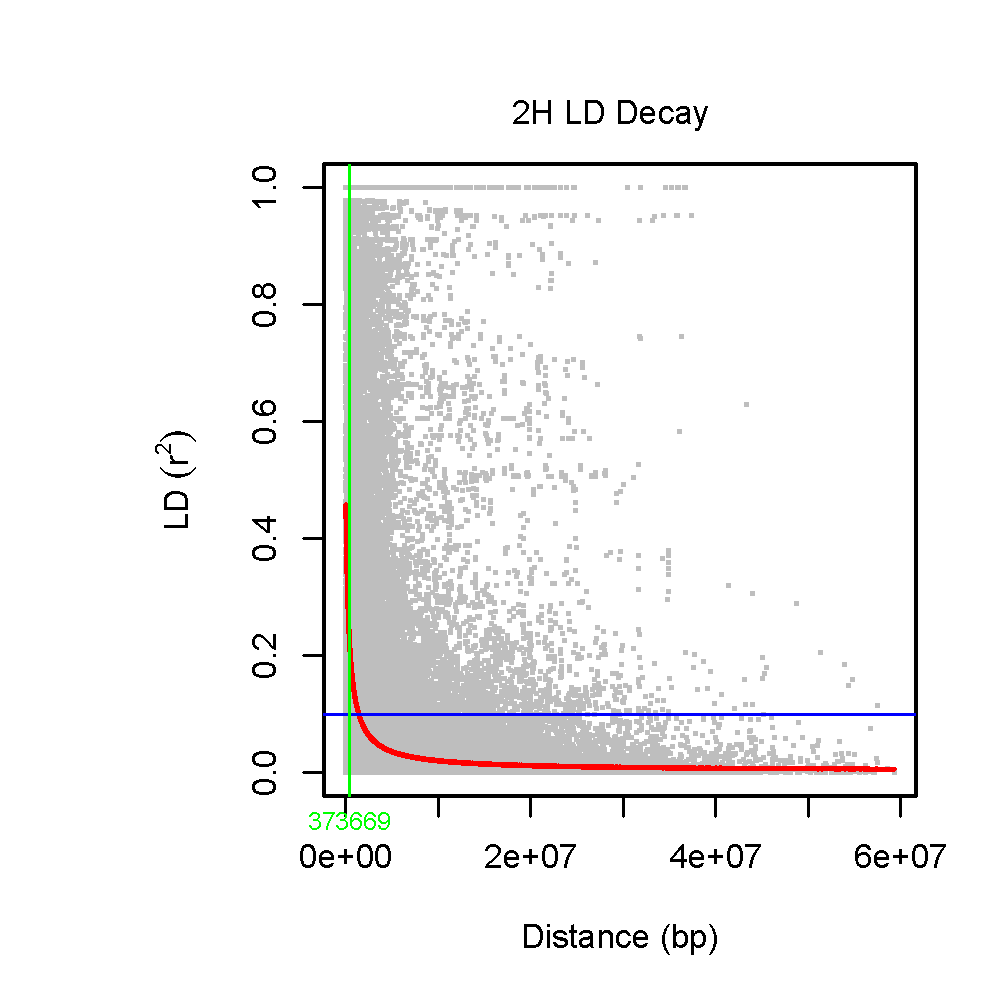

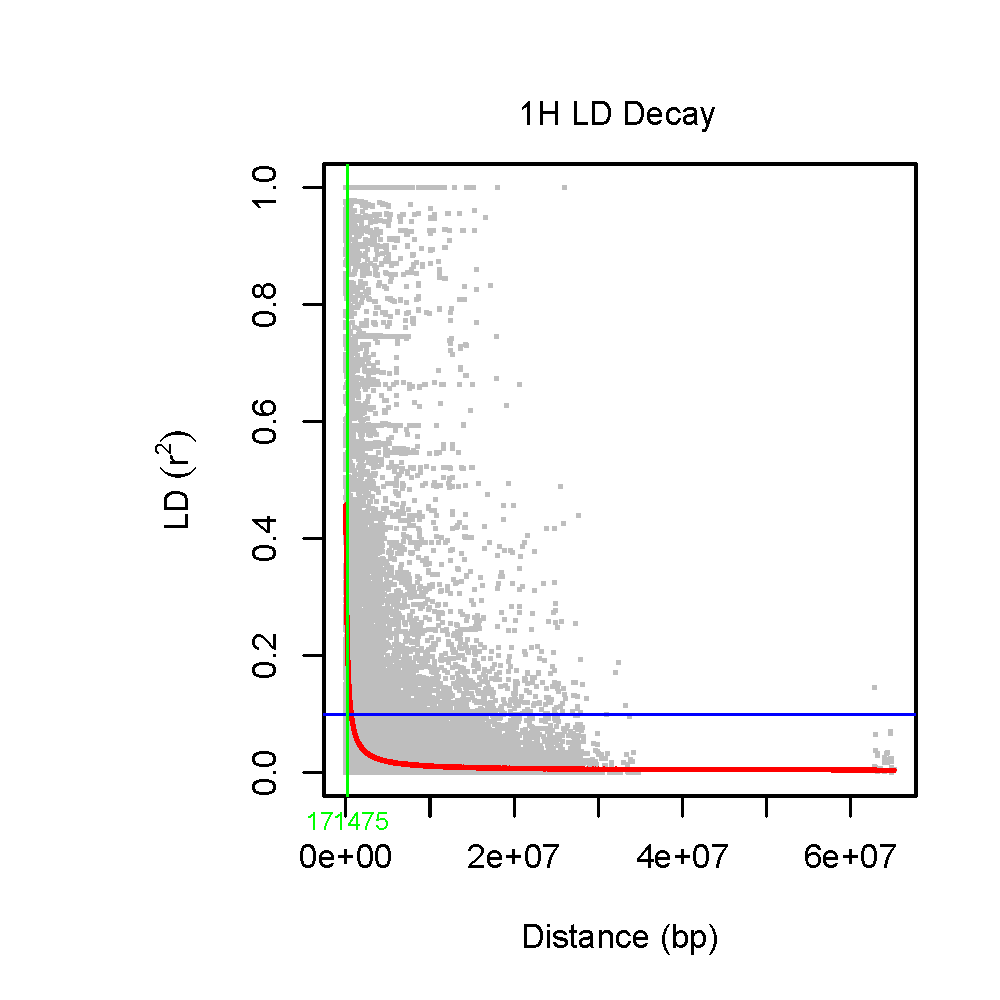


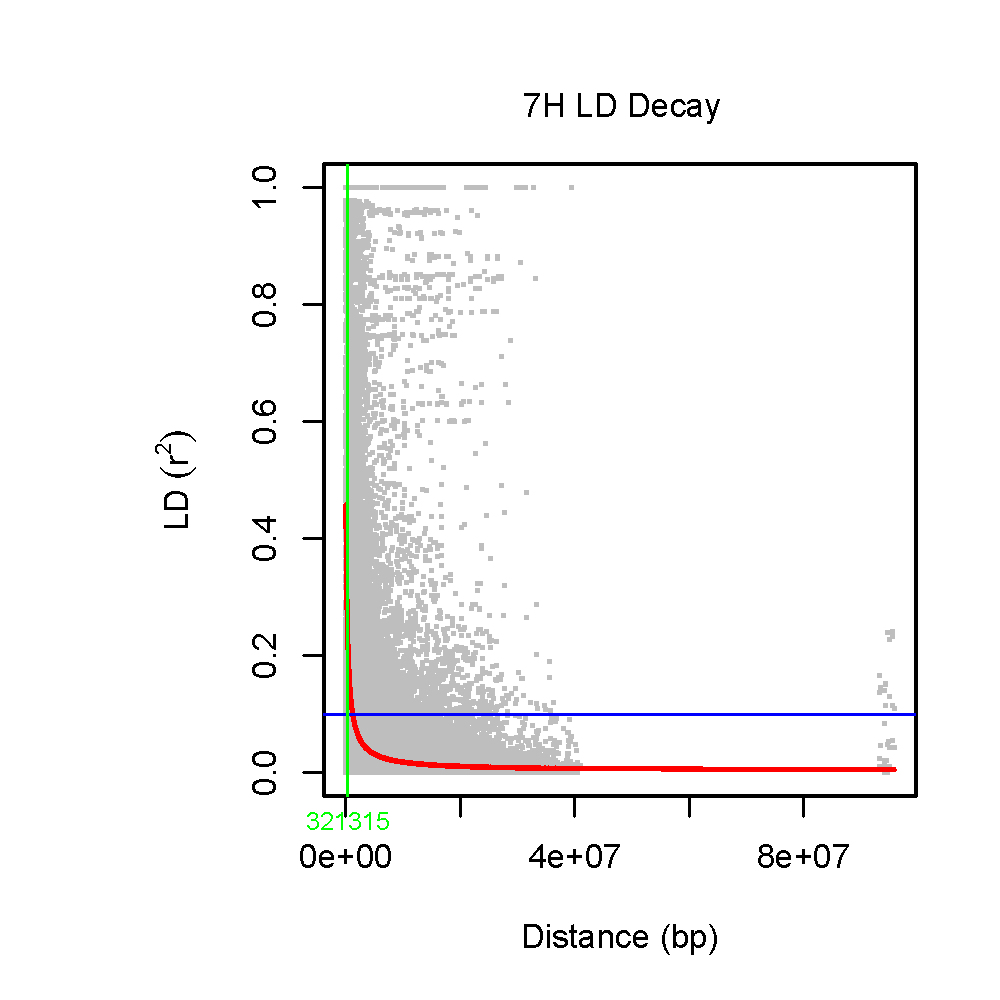

Supplement: Supplementary Figure 1 — Heterozygosity among 191 genotypes and GBS-SNPs used in the current study. The values on the X-axis indicate heterozygous allele proportions ranging from 0 to 1 in the association mapping panel. [file DataSheet_1.zip › Suppl Figures/Suppl Figure 4.docx]

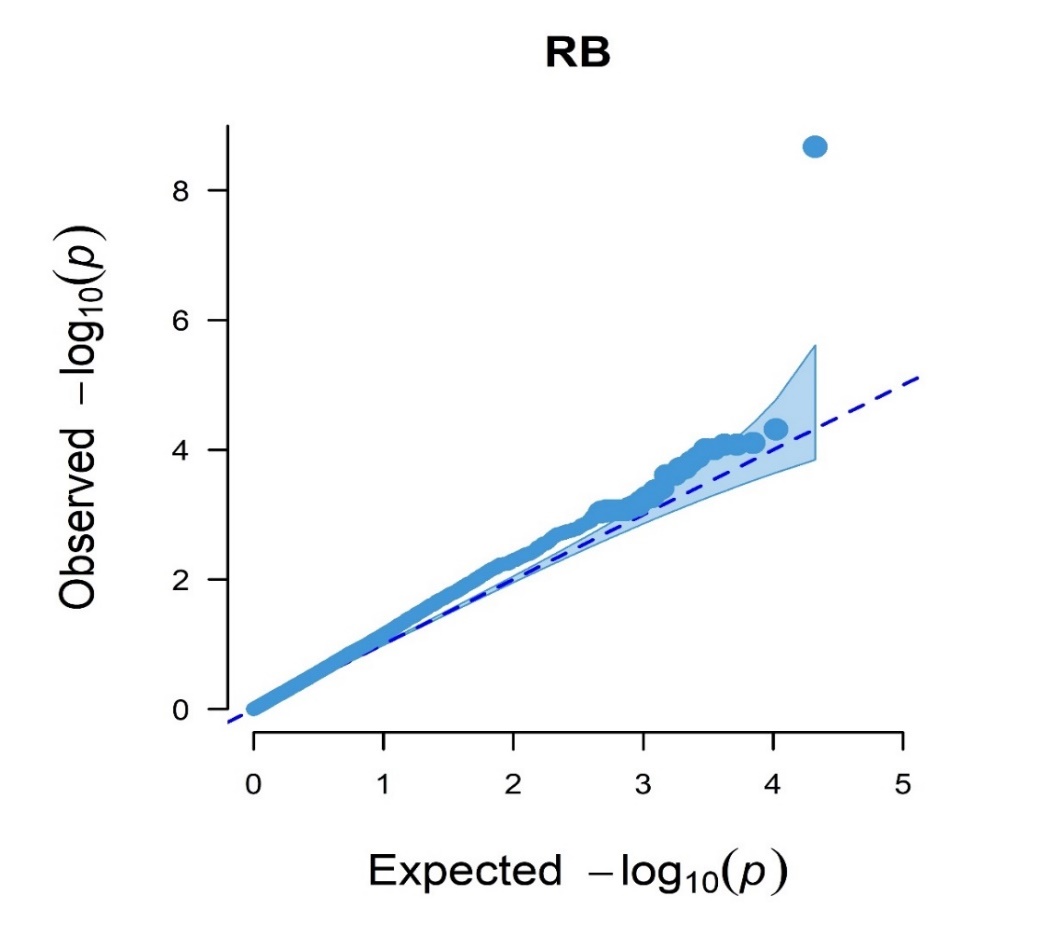


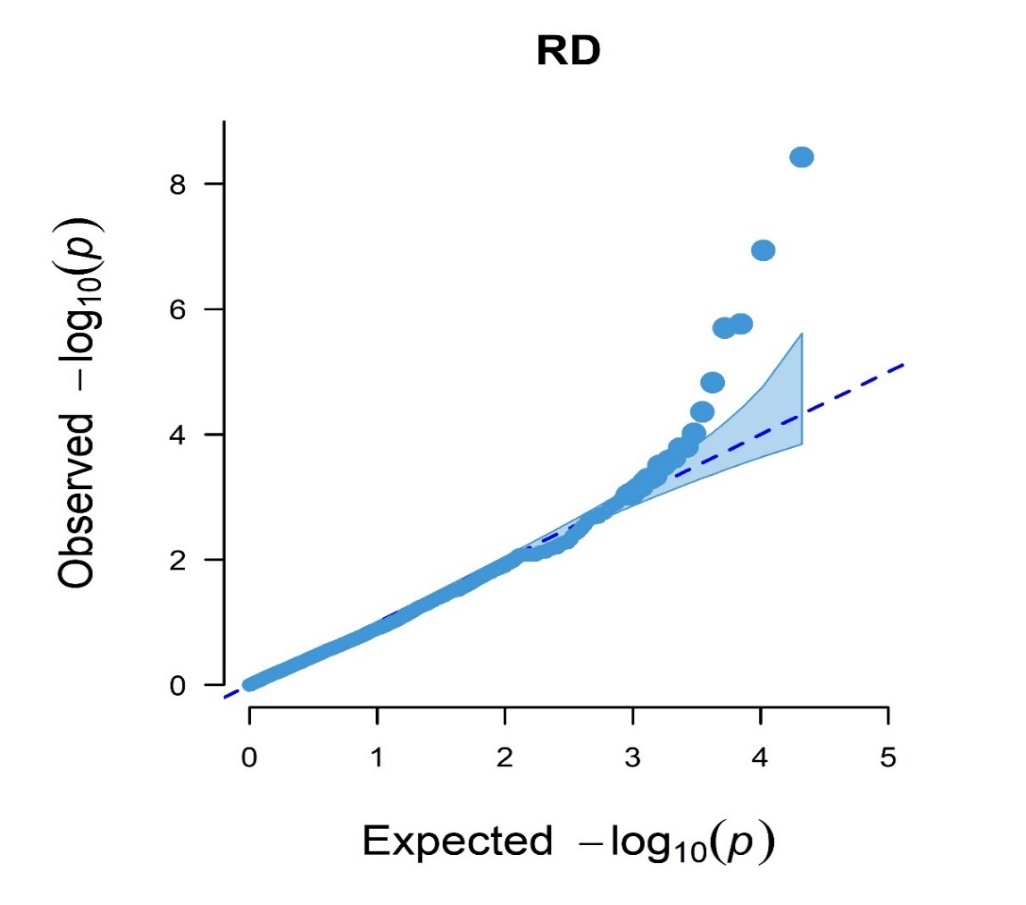


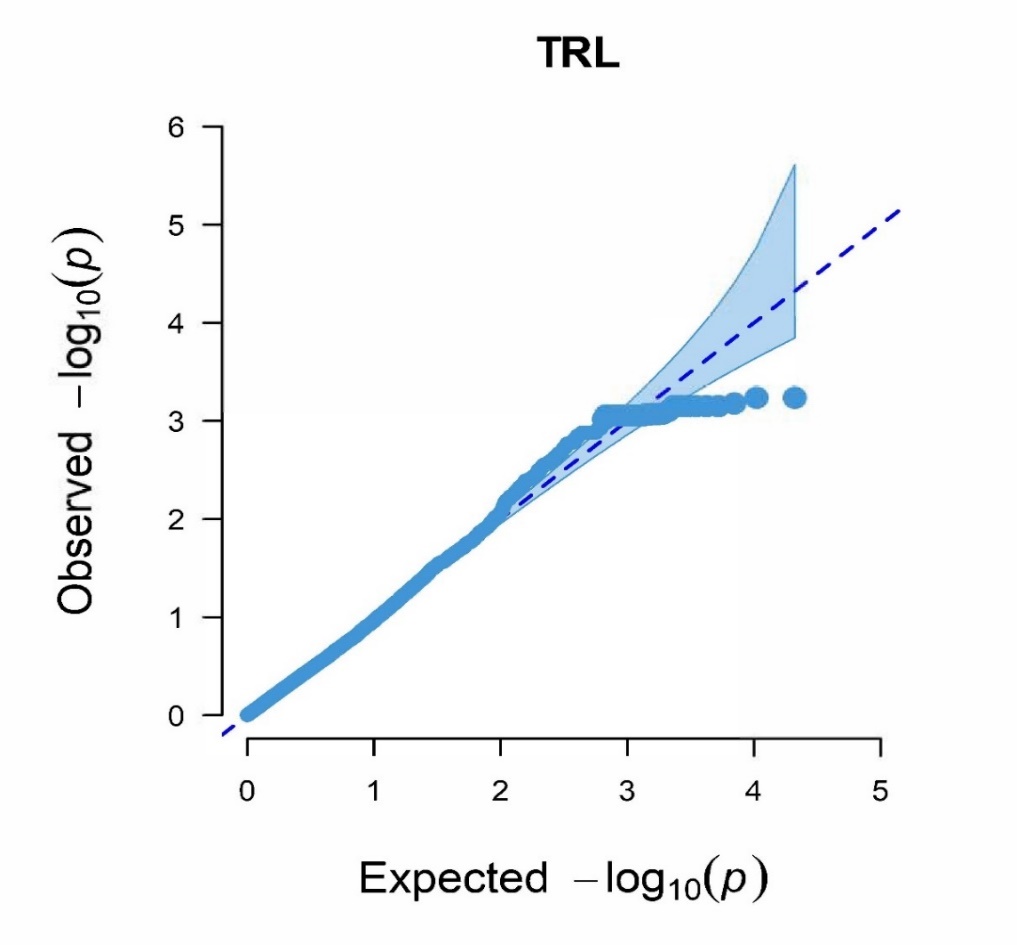


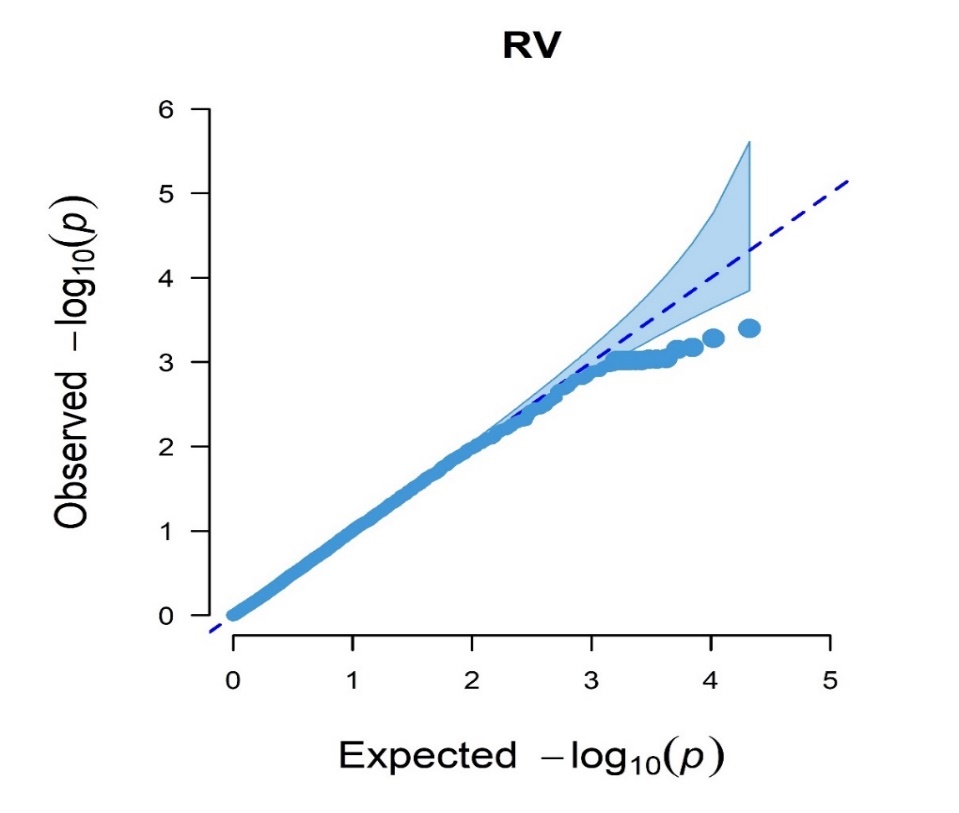


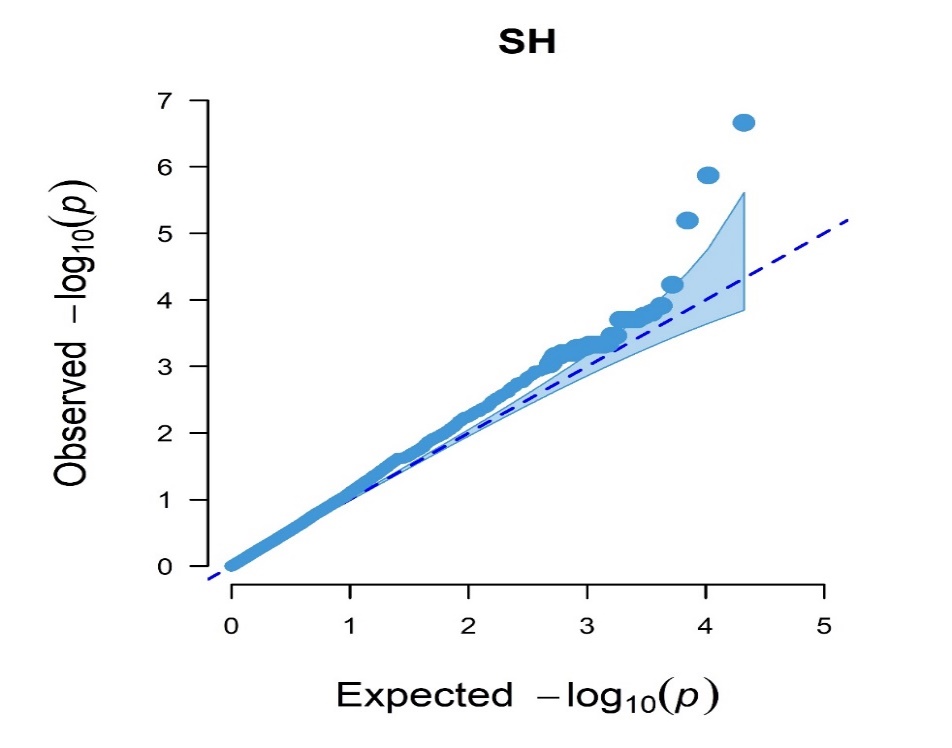

Supplement: Supplementary Figure 1 — Heterozygosity among 191 genotypes and GBS-SNPs used in the current study. The values on the X-axis indicate heterozygous allele proportions ranging from 0 to 1 in the association mapping panel. [file DataSheet_1.zip › Suppl Figures/Suppl Figure 5.docx]
